# Constructing dual charge-transfer tunnels within highly charge-confined COFs for efficient photosynthesis of hydrogen peroxide from water and air

**DOI:** 10.1093/nsr/nwaf444

**Published:** 2025-10-17

**Authors:** Yanghui Hou, Fuyang Liu, Zhengmao Li, Jialiang Liang, Peng Zhou, Meiping Tong

**Affiliations:** College of Environmental Sciences and Engineering, Peking University, Beijing 100871, China; The Key Laboratory of Water and Sediment Sciences (Ministry of Education), Peking University, Beijing 100871, China; State Environmental Protection Key Laboratory of All Material Fluxes in River Ecosystems, Peking University, Beijing 100871, China; College of Environmental Sciences and Engineering, Peking University, Beijing 100871, China; The Key Laboratory of Water and Sediment Sciences (Ministry of Education), Peking University, Beijing 100871, China; State Environmental Protection Key Laboratory of All Material Fluxes in River Ecosystems, Peking University, Beijing 100871, China; College of Environmental Sciences and Engineering, Peking University, Beijing 100871, China; The Key Laboratory of Water and Sediment Sciences (Ministry of Education), Peking University, Beijing 100871, China; State Environmental Protection Key Laboratory of All Material Fluxes in River Ecosystems, Peking University, Beijing 100871, China; College of Environment and Ecology, Chongqing University, Chongqing 400045, China; Eco-environment and Resource Efficiency Research Laboratory, School of Environment and Energy, Peking University Shenzhen Graduate School, Shenzhen 518055, China; College of Environmental Sciences and Engineering, Peking University, Beijing 100871, China; The Key Laboratory of Water and Sediment Sciences (Ministry of Education), Peking University, Beijing 100871, China; State Environmental Protection Key Laboratory of All Material Fluxes in River Ecosystems, Peking University, Beijing 100871, China

**Keywords:** charge-transfer tunnel, charge-separation efficiency, covalent organic frameworks, water-oxidation reaction, H_2_O_2_ photosynthesis

## Abstract

Insufficient charge separation and sluggish two-electron water-oxidation reaction are two critical factors restricting the photosynthesis performance of metal-free covalent organic frameworks (COFs) for hydrogen peroxide (H_2_O_2_) generation from naturally abundant water and air. Herein, we develop a facile strategy to simultaneously boost the charge-separation efficiency and water-oxidation capability through constructing short and rapid charge-transfer tunnels within highly charge-confined COFs via replacing the phenyl with pyrimidine. Compared with a single charge-transfer tunnel within a lowly charge-confined COF-5-(4-aminophenyl)pyrimidin-2-amine (APM) with pyrimidine, dual charge-transfer tunnels are constructed within a highly charge-confined COF-5,5′-bipyrimidine-2,2′-diamine (BPM) with bipyrimidine due to the ground-state charge transfer between para-carbon and meta-nitrogen, which significantly accelerates the intermolecular charge-transfer process and prevents charge recombination. This strategy also decreases the energy barrier of rate-determining water oxidation in H_2_O_2_ photosynthesis and thus promotes the effective generation of the key *OH intermediates, facilitating the generation of H_2_O_2_ at a production rate of 5521 μmol g^−1^ h^−1^ from water, oxygen and light without sacrificial reagents or additional energy consumption by COF-BPM. Furthermore, COF-BPM can also efficiently produce H_2_O_2_ under broad pH conditions, in widely available real water, on a floatable foam sheet, in a continuous-flow reactor and in a scaled-up reactor by using natural solar light for water decontamination.

## Teaser

This work provides new insights into the design of covalent organic frameworks (COFs) by constructing charge transfer tunnels to boost hydrogen peroxide photosynthesis, and paves the way for practical application of COFs in solar-driven synthesis.

## INTRODUCTION

Hydrogen peroxide (H_2_O_2_), as a green oxidant, has been extensively used in organic synthesis, the pulp industry and environmental remediation with an annual global production of >6 million metric tons [1,2]. More than 95% of H_2_O_2_ is currently produced via the multistep anthraquinone process, which needs massive energy consumption and generates substantial toxic waste [3]. Thus, it is highly urgent to develop facile, efficient and eco-friendly approaches to produce H_2_O_2_. Photocatalytic synthesis of H_2_O_2_ from naturally abundant water/air under sunlight irradiation without using sacrificial agents offers a promising alternative [4,5]. Although H_2_O_2_ can be efficiently generated by using metal-based photocatalysts [6,7], secondary pollution would still be caused due to the release of metal ions during the reaction process. Metal-free photocatalysts [8,9] that can avoid this shortage thus have recently received considerable attention for H_2_O_2_ generation. Among various metal-free photocatalysts, covalent organic frameworks (COFs) possessing a high surface area, ordered structure and tunable composition have been regarded as star materials for H_2_O_2_ photosynthesis [10–12]. Nevertheless, the insufficient charge-separation and sluggish two-electron (2*e*^−^) water-oxidation processes still restrict H_2_O_2_ photosynthesis by COFs from water, air and solar light without using sacrificial agents [5,13].

**Figure 1.**
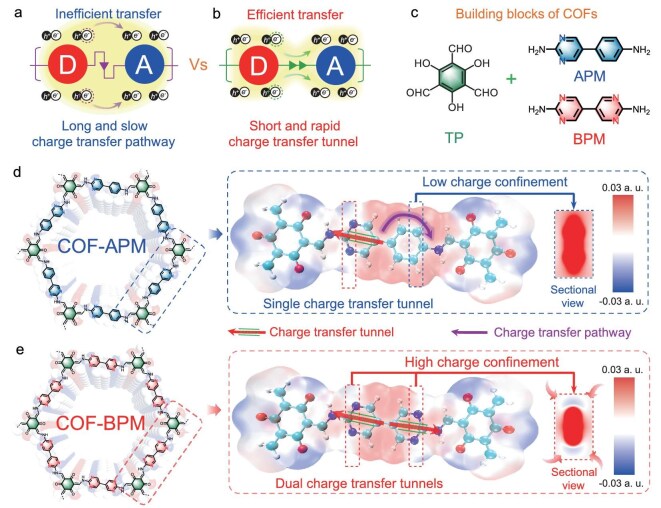
Synthesis and structures of COFs. Schematic representations of (a) the charge-transfer pathway and (b) the tunnel; the D refers to the electron-donor unit while the A refers to the electron-acceptor unit. Structures of (c) building blocks, (d) COF-APM and (e) COF-BPM; the insets show the corresponding electrostatic-potential maps.

To improve the photosynthesis performance of COFs for H_2_O_2_ production, different strategies such as introducing a single atom [14] and post-synthetic modification [15,16] have been utilized to boost the charge separation in COFs. However, these approaches are still time-/energy-consuming owing to the complicated fabrication processes. It is urgent to develop a convenient and efficient strategy to facilitate the charge-separation efficiency of COFs for boosting the production of H_2_O_2_. Modulating the charge-transfer pathway from electron-donor to electron-acceptor units in COFs is a promising and effective strategy to optimize the charge separation and subsequent water-oxidation reaction for promoting H_2_O_2_ photosynthesis [17,18]. Owing to the electron-deficiency nature of nitrogen, increasing the number of nitrogen atoms within the heterocyclic structure of COFs can substantially affect the local charge distribution and regulate the charge-transfer pathways in COFs through ground-state charge-transfer effects, and thus influence the subsequent water-oxidation process [19–21]. The meta-nitrogen atoms in the pyrimidine unit of COFs may effectively confine the positive charge distribution onto carbon atoms. This ground-state charge-transfer process between para-carbon atoms and meta-nitrogen atoms results in a more favorable environment for charge transfer on the donor unit of COFs due to the increased positive charge density [22]. A shorter and faster charge-transfer tunnel between para-carbon atoms relative to the long and slow charge-transfer pathway in phenyl thus can be constructed, which facilitates the charge separation and prevents its recombination (Fig. 1a and b). The subsequent water-oxidation reaction and H_2_O_2_ photosynthesis are expected to be promoted, which, however, has not been explored.

Herein, we demonstrate a facile and effective strategy to boost H_2_O_2_ generation via constructing short and fast charge-transfer tunnels within highly charge-confined COFs via replacing phenyl with pyrimidine (Fig. 1c). Specifically, by integrating a 5-(4-aminophenyl)pyrimidin-2-amine (APM) or 5,5-bipyrimidine-2,2-diamine (BPM), COF-APM (with a single charge-transfer tunnel) and COF-BPM (with dual charge-transfer tunnels) are fabricated via a one-pot solvothermal method. Both COF-APM and COF-BPM possess the ordered crystalline structures, high surface area and excellent visible-light harvesting capacity determined by various characterization techniques. Moreover, the spectroscopy experiments and theoretical calculations demonstrate that COF-BPM simultaneously accelerates the intermolecular charge-transfer process and reduces the energy barrier of rate-determining water oxidation compared to COF-APM, thus achieving a high H_2_O_2_ production rate of 5521 μmol g^-1^ h^-1^ from water, oxygen and light without sacrificial reagents. We further demonstrate that COF-BPM maintains efficient H_2_O_2_ photosynthesis performance under a wide pH range (3–11) and in four kinds of real water samples. Furthermore, COF-BPM can be immobilized onto a floatable foam sheet or a continuous-flow system to stably and effectively generate H_2_O_2_. COF-BPM can also achieve efficient photocatalytic H_2_O_2_ production in an outdoor scaled-up reactor (2 L) under solar irradiation, enabling the effective *ex*  *situ* disinfection of antibiotic-resistant bacteria. Besides, COF-BPM can effectively *in*  *situ* disinfect gram-negative and gram-positive bacteria, as well as degrade emerging organic contaminants. Clearly, the highly charge-confined COF-BPM with dual charge-transfer tunnels has great potential to produce H_2_O_2_ for water decontamination.

## RESULTS AND DISCUSSION

### Material characterizations

**Figure 2.**
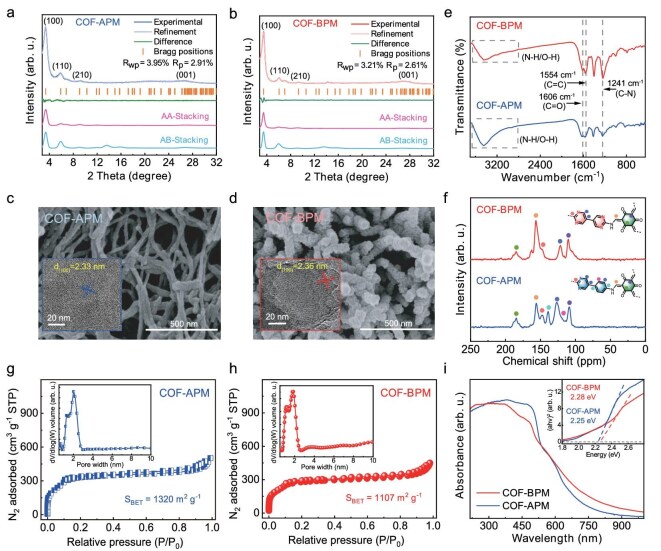
Structural characterizations. PXRD profiles and simulated structures of (a) COF-APM and (b) COF-BPM. Scanning electron microscopy and high-resolution transmission electron microscopy image of (c) COF-APM and (d) COF-BPM. (e) Fourier transform-infrared spectra and (f) ^13^C solid-state nuclear magnetic resonance spectra of the two COFs. N_2_ adsorption–desorption isotherm of (g) COF-APM and (h) COF-BPM; the insets show the corresponding pore-size distribution. (i) Ultraviolet–visible diffuse reflectance spectra patterns of the two COFs; the inset shows the Tauc plots.

Via the condensation between 1,3,5-triformylphloroglucinol (TP, which can form a *β*-ketoenamine structure to enhance the framework stability [23]) and APM or BPM (Fig. 1c), two COFs (named as COF-APM and COF-BPM) with different amounts of pyrimidine were synthesized (Fig. 1d and e). The powder X-ray diffraction (PXRD) analysis confirms the high crystallinity of both COF-APM and COF-BPM owing to the presence of characteristic peaks (Fig. 2a and b). Specifically, the two COFs exhibit similar diffraction peaks at ∼3.4°, 6.0°, 9.1° and 26.8°, corresponding to the (100), (110), (210) and (001) facets, respectively [24]. By using Pawley refinement with an AA-stacking model, the unit cell parameters of COF-BPM are optimized to be a = b = 25.96 Å, c = 3.45 Å, α = β = 90° and γ = 120° with a hexagonal *P*6 space group (*R*_wp_ = 3.04% and *R*_p_ = 2.27%) (Table S1). Similarly, the unit cell parameters of COF-APM are also determined to be a = b = 28.82 Å, c = 3.49 Å, α = β = 90° and γ = 120° with a hexagonal *P*6 space group (*R*_wp_ = 3.44% and *R*_p_ = 2.53%) (Table S2). The scanning electron microscopy results show that COF-APM features a nanowire morphology with a width of ∼40 nm (Fig. 2c), whereas COF-BPM exhibits a nanorod morphology with a width of ∼50 nm (Fig. 2d). Moreover, the high-resolution transmission electron microscopy images exhibit the clear lattice fringes of the (100) facet with a spacing of 2.33 nm for COF-APM (Fig. 2c, inset) and 2.36 nm for COF-BPM (Fig. 2d, inset), further confirming the ordered structure of the two COFs [25].

The Fourier transform-infrared (FTIR) spectra of COF-APM and COF-BPM show the characteristic stretching vibrations at ∼1241, ∼1554 and ∼1606 cm^−1^, which can be attributed to the C–N, C=C and C=O bonds in both COFs, respectively (Fig. 2e) [26,27]. In addition, the signal peaks at ∼109, ∼156 and ∼185 ppm in the solid-state ^13^C nuclear magnetic resonance spectra of COF-APM and COF-BPM correspond to the carbon atoms in the *β*-ketoenamine structure [28], while the peaks at ∼122 and ∼148 ppm for the two COFs correspond to the carbon atoms in the pyrimidine ring (Fig. 2f) [29]_._ The X-ray photoelectron spectroscopy (XPS) results confirm that both COF-APM and COF-BPM are composed of Earth-abundant elements including C, N and O (Fig. S1). Moreover, the N 1s XPS spectra of the two COFs can be resolved into two peaks with binding energies at ∼399 and ∼400 eV, which correspond to N species in the pyrimidine structure and the C–N bond, respectively (Fig. S2) [29]. The thermogravimetric analysis under an N_2_ atmosphere reveals that both COF-APM and COF-BPM are thermally stable (∼450°C) (Fig. S3), indicating the high degree of polymerization for the two COFs. These results demonstrate the successful fabrication of the pyrimidine-based COFs with similar high crystallinity and framework structures.

The Brunauer–Emmett–Teller surface area of COF-APM and COF-BPM are determined to be 1320 and 1107 m^2^ g^−1^, respectively (Fig. 2g and h). By using a nonlocal density functional theory model, the major pore sizes of COF-APM and COF-BPM are calculated to be 1.97 and 1.85 nm, respectively (Fig. 2g and h, inset). The ultraviolet–visible diffuse reflectance spectra (UV–vis DRS) exhibit the wide optical absorption from 400  to 700 nm for both COFs, suggesting their excellent visible-light-harvesting capacity (Fig. 2i). The optical band gaps (*E*_g_) of COF-APM and COF-BPM are estimated to be 2.25 and 2.28 eV, respectively, from the Tauc plot (Fig. 2i, inset). According to the valence-band XPS analysis, the valence-band positions (*E*_VB_) of the two COFs are determined to be 1.89 and 1.88 V vs. normal hydrogen electrode (NHE), respectively (Fig. S4a) [30]. The corresponding conduction-band positions (*E*_CB_) of COF-APM and COF-BPM are further deduced to be −0.36 and −0.39 V vs. NHE, respectively. The energy-level diagrams of COF-APM and COF-BPM are provided in Fig. S4b, which clearly indicates that the redox potentials of *E*_VB_ and *E*_CB_ for both COFs are thermodynamically favorable for a photocatalytic stepwise 2*e*^−^ oxygen-reduction reaction (ORR) and 2*e*^−^ water-oxidation reaction (WOR) to generate H_2_O_2_ without the need for sacrificial agents [31].

**Figure 3.**
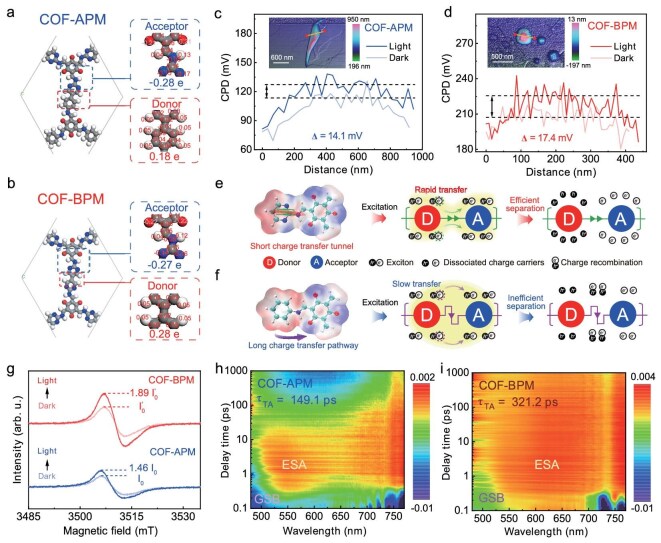
Charge-separation and charge-transfer mechanisms. Hirshfeld charge distribution in (a) COF-APM and (b) COF-BPM. Kelvin probe force microscopy (KPFM) potential profiles of (c) COF-APM and (d) COF-BPM; the dashed line displays the average value of the potentials and the inset shows the corresponding KPFM image. Schematic representation of photo-induced charge-transfer processes for (e) charge-transfer tunnel and (f) pathway. (g) *In*  *situ* ESR spectra of the two COFs. 2D TA spectra of (h) COF-APM and (i) COF-BPM.

The Hirshfeld charge analysis of COF-APM and COF-BPM shows that the electron-rich pyrimidine C and benzene unit in both COFs serve as the electron-donor structures, whereas the electron-deficient pyrimidine N and *β*-ketoenamine structure in both COFs serve as the electron-acceptor structures (Fig. 3a and b) [32]. Moreover, the total donor charge (0.28 e) within COF-BPM is greater than that within COF-APM (0.18 e), which illustrates that, compared with COF-APM, COF-BPM with the optimal local charge distribution has a stronger electron-donating capability to boost the intermolecular charge-transfer process [33]. The surface electrostatic potentials (ESP) show that the positive ESP regions of COF-APM are distributed on carbon atoms within both the pyrimidine and benzene structure, while the positive ESP regions of COF-BPM are only distributed on carbon atoms within pyrimidine units through the ground-state charge-transfer effects (Fig. 1d and e, inset) [22]. Clearly, the meta-nitrogen atoms in the pyrimidine unit of COFs can confine the positive charge distribution on carbon atoms, which indicates that the photo-induced electron can only be generated from the carbon atoms in the pyrimidine unit and then transferred to the *β*-ketoenamine structure through the shorter charge-transfer tunnel between the para-carbon atoms. Thus, one short charge-transfer tunnel plus one long charge-transfer pathway are present within the lowly charge-confined COF-APM, whereas dual charge-transfer tunnels between the electron-donor and electron-acceptor unit exist within the highly charge-confined COF-BPM containing the more confined local charge distribution (Fig. 1d and e, inset). The Kelvin probe force microscopy results show that the surface potential of COF-BPM (∼17.4 mV) is higher than that of COF-APM (∼14.1 mV) after light irradiation (Fig. 3c and d), verifying the enhanced self-driven migration of photogenerated charges in COF-BPM relative to COF-APM [34].

The *in*  *situ* irradiated XPS analysis of COF-APM and COF-BPM exhibits that the binding energy of N 1s (including pyrimidine N and amine N) for both COFs shifts towards the positive direction after irradiation (Fig. S5), which demonstrates that the photogenerated electrons are generated by the pyrimidine/benzene unit and transfer to the *β*-ketoenamine structure in both COFs [35]. The charge-transfer processes in different charge-confined COFs at the molecular level were then investigated based on theoretical calculations. The excited-state charge distribution suggests that the short charge-transfer tunnel (10.03 arbitrary units (arb. u.)) has a larger charge delocalization index than the long charge-transfer pathway (8.75 arb. u.) (Fig. S6), confirming the more effective intermolecular charge-transfer process in the short tunnel relative to the long pathway (Fig. 3e and f) [36]. The *in*  *situ* electron spin resonance (ESR) spectra results exhibit that COF-BPM has a stronger ESR signal than COF-APM after irradiation (Fig. 3g), which further confirms that COF-BPM with dual charge-transfer tunnels contains more free charges than COF-APM with a single charge-transfer tunnel. The transient photocurrent measurement and electrochemical impedance spectroscopy exhibit that COF-BPM contains a higher photocurrent density (Fig. S7a) and smaller semicircle relative to COF-APM (Fig. S7b), suggesting more efficient charge-carrier separation and reduced charge-transfer resistance in COF-BPM compared with COF-APM. Photoluminescence (PL) analysis also shows that the emission intensity of COF-BPM is lower than that of COF-APM (Fig. S8a), suggesting the inhibited recombination of photogenerated charge in COF-BPM relative to COF-APM. Furthermore, the average fluorescent lifetime of COF-BPM (0.39 ns) is also lower than that of COF-APM (0.52 ns) (Fig. S8b), indicating the faster charge-transfer dynamics of COF-BPM than COF-APM [37]. Clearly, the above results verify that, compared with COF-APM containing a single charge-transfer tunnel, COF-BPM containing dual charge-transfer tunnels facilitates the intermolecular charge-transfer process and prevents charge recombination, which thus can generate more free charges during the photocatalytic process for H_2_O_2_ production.

The exciton binding energy (*E*_b_)—a critical factor to illustrate the interaction force of excitons (bound electron–hole pairs)—was investigated via temperature-dependent PL spectroscopy [38]. The *E*_b_ values of COF-APM and COF-BPM are determined to be 85.6 and 75.8 meV, respectively, which suggests that the exciton in COF-BPM can be more easily dissociated into free charges than that in COF-APM (Fig. S9). Femtosecond transient absorption (fs-TA) spectra were conducted to investigate the dynamics of photoexcited carriers within the COFs. After the photoexcitation, the fs-TA spectra of both COFs show a significant signal between 475 and 525 nm with negative changes in absorbance, corresponding to the ground-state bleaching signal (Fig. 3h and i) [39]. Besides, a broad positive signal at 525–700 nm in both COFs is also detected, attributed to the excited-state absorption (ESA) signal. Furthermore, the ESA signal of COF-BPM at the picosecond level is much stronger relative to that of COF-APM (Fig. S10), which suggests that COF-BPM contains more photogenerated carriers under photoexcitation than does COF-APM [10]. Based on the TA decay kinetic analysis, the excited-state lifetime of COF-BPM is estimated to be 321.2 ps, which is 2.2 times longer than that of COF-APM (149.1 ps) (Fig. S11 and Table S3). This indicates the more efficient exciton dissociation of COF-BPM relative to COF-APM [40]. All of the above results confirm that, compared with the lowly charge-confined COF-APM with a single charge-transfer tunnel, the intermolecular charge-transfer process in the highly charge-confined COF-BPM with dual charge-transfer tunnels is greatly accelerated, which can facilitate the injection of photo-induced charge into H_2_O and O_2_ to boost H_2_O_2_ photosynthesis (discussed in detail below).

### Photocatalytic performance

**Figure 4.**
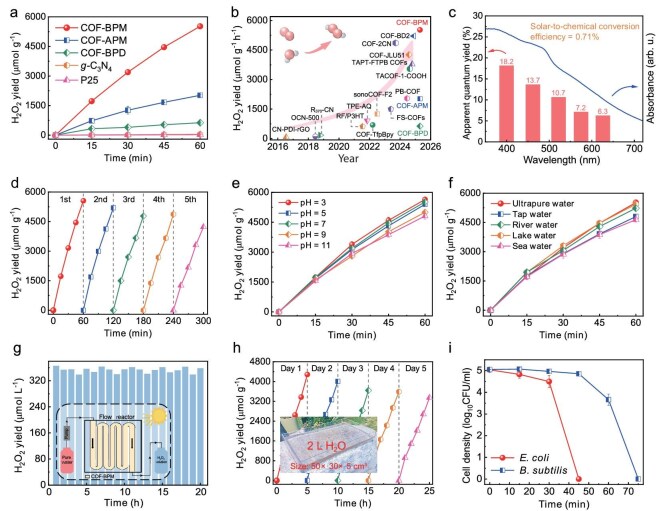
Photocatalytic H_2_O_2_-production performance. (a) H_2_O_2_ photosynthesis rates of different photocatalysts. (b) Comparison of H_2_O_2_-production rates by COF-BPM with other photocatalysts. (c) Apparent quantum yield of COF-BPM. (d) Cycling performance of COF-BPM for H_2_O_2_ production. (e) Effects of initial solution pH on H_2_O_2_ photosynthesis rates by COF-BPM. (f) H_2_O_2_ photosynthesis rates by COF-BPM in ultrapure water, tap water, river water, lake water and seawater. Photosynthesis of H_2_O_2_ by immobilized COF-BPM (g) in a continuous-flow system for 20 h and (h) in an outdoor scaled-up reactor under solar irradiation for 5 sunny days; the insets show the digital images of different reactors. (i) *In*  *situ* photocatalytic disinfection performance by COF-BPM. General conditions: λ > 420 nm (298 K; xenon lamp, light intensity: 100 mW cm^−2^), ultrapure water (40 mL), photocatalyst (5 mg). Error bars represent the standard deviation and are calculated on the basis of two independent experiments.

The photosynthetic performance of COF-APM and COF-BPM for H_2_O_2_ generation from pure water and oxygen is evaluated under visible-light irradiation without sacrificial agents. COF-APM with a single charge-transfer tunnel can generate H_2_O_2_ at a production rate of 2023 μmol g^−1^ h^−1^ (Fig. 4a and Fig. S12), surpassing the biphenyl-based COF (COF-BPD, 631 μmol g^−1^ h^−1^) containing two long charge-transfer pathways yet without a charge-transfer tunnel under the same conditions (Fig. S13) [41]. Notably, COF-BPM with dual charge-transfer tunnels can produce H_2_O_2_ at a remarkable production rate of 5521 μmol g^−1^ h^−1^, which is 2.73 times higher than that of COF-APM with a single charge-transfer tunnel (Fig. 4a and Fig. S12). In addition, the H_2_O_2_ photosynthesis performance of COF-BPM exceeds that of traditional photocatalysts (P25 and *g*-C_3_N_4_) and the majority of similar photocatalysts (Fig. 4a and b, and Table S4). These results suggest that the additional charge-transfer tunnels constructed within COFs via replacing phenyl with pyrimidine can significantly enhance the photocatalytic activity for H_2_O_2_ generation. The results of two additional control groups of COFs also confirm that the additional charge-transfer tunnel constructed within charge-confined COFs via replacing phenyl with pyrimidine can improve the photocatalytic activity of COFs (Fig. S14 and Table S5). Clearly, constructing additional charge-transfer tunnels within COFs by simply replacing phenyl with pyrimidine is a facile and cost-effective strategy to boost H_2_O_2_ photosynthesis.

The H_2_O_2_ decomposition test reveals that the side reaction between COF-BPM and H_2_O_2_ is negligible, contributing to the efficient and stable H_2_O_2_ production (Fig. S15). Moreover, compared with the high H_2_O_2_ formation rate constants (*K*_f_, 15.56 and 16.86 μM min^−1^, respectively, at 25°C and 40°C), the H_2_O_2_ decomposition rate constant of COF-BPM is low (*K*_d_, 10.60 × 10^−3^ and 11.25 × 10^−3^ min^−1^, respectively, at 25°C and 40°C), which also leads to the efficient H_2_O_2_-production rate (Fig. S16) [42]. Due to the enhancement of photon absorption by COF particles [43], decreasing the dosages of COFs in reaction systems can significantly improve the H_2_O_2_ photosynthesis performance (Fig. S17). Notably, the H_2_O_2_ yield of COF-BPM can reach 8446 μmol g^−1^ h^−1^ by adding 1 mg of COFs into 40 mL of pure water, which is also higher than that produced by COF-APM (3384 μmol g^−1^ h^−1^). Moreover, COF-BPM exhibits an impressive apparent quantum yield of 18.2% at 400 nm with a high solar-to-chemical conversion efficiency of 0.71% (Fig. 4c). The reuse experiments show that the excellent H_2_O_2_-production rate of COF-BPM can be achieved even after five consecutive reused cycles (Fig. 4d). PXRD analysis (Fig. S18a), FTIR spectra (Fig. S18b), N_2_ adsorption–desorption isotherms (Fig. S18c), and UV–vis DRS spectra (Fig. S18d) suggest that the crystallinity and structure of COF-BPM do not change significantly after five consecutive cycles, confirming the excellent stability of COF-BPM during the photocatalytic H_2_O_2_ production. Additionally, COF-BPM can efficiently generate H_2_O_2_ in water with a broad pH range (3–11), showing its high tolerance to the different solution pH values (Fig. 4e and Fig. S19). Owing to the presence of dissolved organic matter and coexisting ions in actual water matrices (Table S6), the H_2_O_2_ photosynthesis performances in four actual water matrices are slightly decreased in comparison with that in pure water (Fig. 4f and Fig. S20). Nevertheless, the high H_2_O_2_ yield by COF-BPM can still be achieved in tap water (4797 μmol g^−1^ h^−1^), river water (5232 μmol g^−1^ h^−1^), lake water (5434 μmol g^−1^ h^−1^) and seawater (4641 μmol g^−1^ h^−1^), verifying the feasibility of COF-BPM for H_2_O_2_ generation by using different types of actual water.

To ease reuse, COF-BPM particles were fixed onto a floatable foam sheet for H_2_O_2_ production; ∼400 μmol L^−1^ of H_2_O_2_ solution after a 1-h reaction can be generated even in the fifth reuse cycle (Fig. S21). Moreover, COF-BPM powders can be incorporated into a continuous-flow system for the continuous production of H_2_O_2_ (Fig. 4g, inset). Under visible-light irradiation, ∼350 μmol L^−1^ of H_2_O_2_ can be generated throughout an entire 20-h reaction period in a continuous reactor system (Fig. 4g). In addition, ∼400 μmol L^−1^ of H_2_O_2_ solution can be generated within 5 h under natural solar irradiation in an outdoor scaled-up reactor (50 × 30 × 5 cm^3^, 2-L working volume) with the immobilized COF-BPM powders, which remains stable over 5 consecutive sunny days (Fig. 4h and Fig. S22). These results confirm that COF-BPM has great application potential for the large-scale production of H_2_O_2_. The produced H_2_O_2_ solution in the scaled-up reactor can effectively *ex*  *situ* disinfect kanamycin-resistant *Escherichia coli* (Fig. S23). Furthermore, COF-BPM can also efficiently *in*  *situ* disinfect gram-negative *E. coli* and gram-positive *Bacillus subtilis* (Fig. 4i), as well as decontaminate emerging organic contaminant (paracetamol) under visible-light irradiation (Fig. S24). The above results clearly demonstrate that highly charge-confined COF-BPM with dual charge-transfer tunnels has great potential to photosynthesize H_2_O_2_ in water with a broad pH range, in different types of real water, on a floatable foam sheet, in a continuous-flow reactor and in a scaled-up reactor using solar light for water decontamination.

### Photocatalytic mechanism

**Figure 5.**
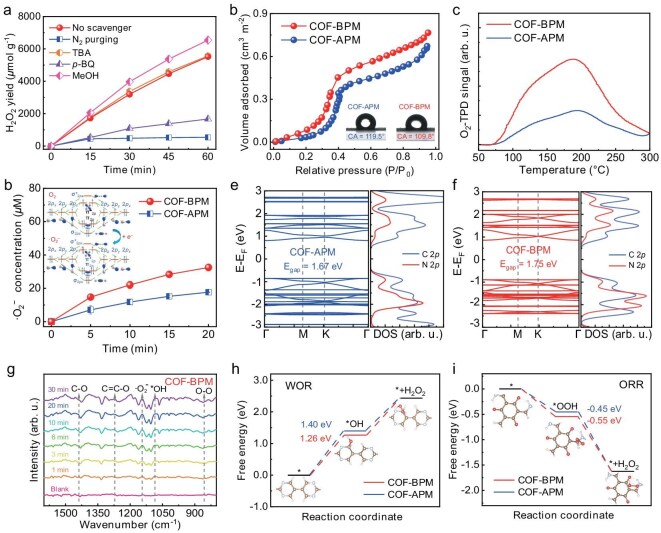
Photocatalytic mechanisms for H_2_O_2_ production. (a) Control experiments of H_2_O_2_ photosynthesis by COF-BPM. (b) Water-adsorption isotherms of the two COFs; the insets show the corresponding water-contact angles. (c) O_2_-temperature-programmed desorption curves of the two COFs. (d) Nitro blue tetrazolium measurements of the two COFs; the inset shows the molecular orbital energy levels and electron arrangement of O_2_ and ⋅O_2_^−^. Energy-band structures and projected density of states (DOS) of (e) COF-APM and (f) COF-BPM. (g) Time-course *in situ* diffuse reflectance infrared Fourier transform spectroscopy (DRIFTS) of COF-BPM. Energy profiles of (h) the water-oxidation path and (i) the oxygen-reduction path in the two COFs for H_2_O_2_ generation. Error bars represent the standard deviation and are calculated on the basis of two independent experiments.

When replacing an O_2_ atmosphere with N_2_, the H_2_O_2_ photosynthesis yield of COF-BPM significantly dropped (Fig. 5a and Fig. S25), which suggests that oxygen is involved in the photosynthesis process of H_2_O_2_. A photocatalytic oxygen evolution experiment with the presence of NaBrO_3_ (as a trapping agent for photogenerated *e*^−^) reveals that COF-BPM is unable to produce O_2_ via the 4*e*^−^ WOR pathway (Fig. S26). The addition of tert-butyl alcohol (as a quenching agent for diffusing ·OH) to the photocatalytic system has no effect on the H_2_O_2_-production performance of COF-BPM (Fig. 5a and Fig. S25), indicating the negligible contribution of ·OH to H_2_O_2_ production. The absence of the diffusing ·OH in the ESR spectra of COF-BPM after irradiation (Fig. S27) further confirms that diffusing ·OH is not involved in the photosynthesis process of H_2_O_2_ [13]. The isotope-labeling experiment of COF-BPM by using oxygen-18-labeled water indicates the involvement of the 2*e*^−^ WOR pathway in H_2_O_2_ production (Fig. S28), which implies that the adsorbed *OH is rapidly oxidized to form H_2_O_2_ [44,45]. The ratio of the contribution of ORR and WOR to H_2_O_2_ generation is calculated to be 1.27:1, which is close to the ratio of 1:1. After methanol (MeOH, as a trapping agent for *h*^+^) is added into the photocatalytic system, the H_2_O_2_ yield of COF-BPM is slightly increased due to the enhanced ORR process (Fig. 5a and Fig. S25) [46]. This suggests that the WOR process is the rate-determining step in the H_2_O_2_ photosynthesis [47,48]. When *p*-benzoquinone (*p*-BQ, as a quenching agent for ·O_2_^–^) is added, the H_2_O_2_-production rate decreases dramatically, indicating the indispensable role of ·O_2_^−^ in the ORR process (Fig. 5a and Fig. S25). The presence of an ·O_2_^−^ signal in the ESR spectra of COF-BPM after irradiation (Fig. S29) further confirms that ·O_2_^−^ makes a contribution to the production of H_2_O_2_. The rotating ring-disk electrode measurement of COF-BPM shows that the average electron-transfer number is 2.66 (Fig. S30), which demonstrates that COF-BPM is more favorable for the 2*e*^−^ ORR pathway to generate H_2_O_2_. Clearly, the H_2_O_2_-production path of COF-BPM is a dual-channel mechanism through the 2*e*^−^ WOR and stepwise 2*e*^−^ ORR process [47,49].

The water-adsorption isotherms of COF-APM and COF-BPM show that the water-adsorption capacity of COF-BPM is greater than that of COF-APM (Fig. 5b), which is consistent with the result of the water-contact-angle experiment (Fig. 5b, inset). This phenomenon suggests that COF-BPM with stronger water affinity is more favorable for water activation in the H_2_O_2_ photosynthesis process than is COF-APM. The temperature-programmed desorption analysis of O_2_ reveals that the O_2_ adsorption capacity of COF-BPM is greater than that of COF-APM (Fig. 5c). Moreover, the calculated adsorption energy of O_2_ onto COF-BPM (−0.18 eV) is lower than that onto COF-APM (−0.13 eV) (Fig. S31). This confirms that, compared with the lowly charge-confined COF-APM, O_2_ is prone to be adsorbed onto the highly charge-confined COF-BPM, contributing to the superior O_2_ adsorption capacity of COF-BPM [39]. Owing to the presence of an unfilled anti-bonding orbital (π*_2p_) of O_2_, the photogenerated *e*^−^ of COFs can inject into the π*_2p_ orbital of O_2_ to generate ·O_2_^−^ through a single electron-transfer process (Fig. 5d, inset) [4]. As expected, the nitro blue tetrazolium reduction measurement exhibits that the formation rate of the ·O_2_^−^ of COF-BPM is larger than that of COF-APM (Fig. 5d), further confirming the higher electron-injection capacity in COF-BPM for ·O_2_^−^ generation relative to COF-APM. Clearly, the above results demonstrate that the highly charge-confined COF-BPM with dual charge-transfer tunnels possesses higher water-oxidation and oxygen-activation capacity than the lowly charge-confined COF-APM with a single charge-transfer tunnel. The calculated energy band exhibits that COF-BPM contains more electron density in the valence band near the Fermi level than COF-APM, which indicates that the photogenerated *e*^−^ in COF-BPM can be more easily excited relative to that in COF-APM (Fig. 5e and f). The projected density of states further reveals that the valence-band top and conduction-band bottom for COF-APM and COF-BPM are primarily contributed by the C 2p orbital, which implies that C atoms in both COFs serve as the active centers during the WOR and ORR processes (Fig. 5e and f, inset).

The *in*  *situ* diffuse reflectance infrared Fourier transform spectroscopy (DRIFTS) results show that the vibration signals of C–OH (1086 cm^−1^) and C=C–O (1271 cm^−1^) are observed for both COFs (Fig. 5g and Fig. S32), which confirms that *OH and O_2_ are adsorbed on the C atom within both COFs, respectively [50,51]. Additionally, the intensity of vibration signals for O–O (867 cm^−1^) and ·O_2_^−^ (1144 cm^−1^) are increased under visible-light irradiation for both COFs (Fig. 5g and Fig. S32), which demonstrates that the adsorbed oxygen is activated by photogenerated *e*^–^ to generate ·O_2_^−^ [52]. The Gibbs free energies (ΔG) based on the 2*e*^−^ WOR and 2*e*^−^ ORR processes for H_2_O_2_ production by COF-APM and COF-BPM were then investigated. In the 2*e*^−^ WOR process, COF-BPM exhibits lower energy barriers (1.26 eV) for H_2_O dehydrogenation to form *OH than does COF-APM (1.40 eV) (Fig. 5h), which indicates that, compared with the pyrimidine in COF-APM, the bipyrimidine in COF-BPM is more favorable for the generation of *OH, facilitating the generation of H_2_O_2_ through the 2*e*^−^ WOR path [53]. In the 2*e*^−^ ORR process, the energy change of *OOH intermediate for COF-BPM (−0.55 eV) is also less than that for COF-APM (−0.45 eV) (Fig. 5i), which demonstrates that O_2_ on COF-BPM is beneficial for the formation of the *OOH intermediate to generate H_2_O_2_ via the 2*e*^−^ ORR path relative to COF-APM. Due to the uphill thermodynamics in the 2*e*^−^ WOR process, the highly charge-confined COF-BPM with enhanced charge transfer exhibits a lower energy barrier in the rate-determining WOR process than does the lowly charge-confined COF-APM. A superior H_2_O_2_-production rate thus is achieved by COF-BPM compared with COF-APM. Based on the theoretical analysis and experimental results, the H_2_O_2_-production mechanism of COF-BPM is provided in Fig. S33. Specifically, in the 2*e*^−^ WOR process, the H_2_O molecules are oxidized by photogenerated *h*^+^ to generate adsorbed *OH (Supplementary Equation S8), which is subsequently oxidized to produce H_2_O_2_ (Supplementary Equation S9). In the stepwise 2*e*^−^ ORR process, O_2_ molecules are effectively reduced by photogenerated *e*^−^ to generate ·O_2_^−^ (Supplementary Equation S10), which further undergoes protonation to generate H_2_O_2_ (Supplementary Equation S11).

## CONCLUSION

The photocatalytic H_2_O_2_ production by COFs is greatly affected by the charge transfer process and water-oxidation reaction process. Considering these issues, a highly charge-confined COF-BPM with dual charge-transfer tunnels is designed to simultaneously accelerate the intermolecular charge-transfer process and reduce the energy barrier of rate-determining water oxidation compared to the lowly charge-confined COF-APM with a single charge-transfer tunnel. A high H_2_O_2_ production rate (5521 μmol g^-1^ h^-1^) is achieved by merely consuming water, oxygen and light without the need for sacrificial reagents or additional energy input. Furthermore, COF-BPM can produce H_2_O_2_ in water with different solution pH values and in various types of real water samples, thereby significantly reducing the cost of H_2_O_2_ production. Besides, COF-BPM can be fixed onto a floatable foam sheet and immobilized into a continuous-flow reactor and an outdoor enlarged reactor for efficient H_2_O_2_ production, enabling effective *ex situ* bacterial inactivation, which can reduce the operational costs for the recycle and reuse of photocatalysts during practical applications. Clearly, this study not only offers new insights for designing efficient COFs through constructing additional charge-transfer tunnels, but also facilitates the practical application of COF-based materials in H_2_O_2_ photosynthesis for water decontamination.

## METHODS

### Synthesis of COF-APM

COF-APM was fabricated via a one-pot solvothermal method. Specifically, TP (84.1 mg), APM (111.7 mg), 1,4-dioxane (5 mL), mesitylene (5 mL) and 6 M acetic acid (HAc, 1 mL) were placed into a 20-mL Teflon lining and then sonicated for 30 min. Subsequently, the Teflon lining was taken in an autoclave and put into an oven and heated at 120°C for 120 h. After it had cooled to room temperature, the solid powder was collected and washed with copious amounts of water and acetone six times. Finally, the sample was dried at 60°C under air for 18 h.

### Synthesis of COF-BPM

COF-BPM was also fabricated via the same one-pot solvothermal method as described previously for COF-APM, except that BPM was used instead of APM.

### Photosynthesis experiments of H_2_O_2_

The photosynthesis of H_2_O_2_ was first carried out in a double-wall quartz reactor. Typically, 5 mg of COF powder was dispersed into 40 mL of pure water. Subsequently, the reaction system was stirred for 10 min under dark conditions with O_2_ as a purging gas. During the photocatalytic process, the reaction system was irradiated by using a 300-W Xenon lamp with a filter (λ > 420 nm, 100 ± 1 mW cm^−2^) and the temperature of the reaction system was fixed at 25.0 ± 0.2°C. For detection of the H_2_O_2_ yield, 1.0 mL of suspension was extracted and filtered every 15 min. To explore the possibility of fabricated COFs in practical application, H_2_O_2_ photosynthesis by COF-BPM in water with a broad pH range, in actual water, on a floatable foam sheet, in a continuous-flow system and in an outdoor scaled-up reactor was also conducted. Especially, H_2_O_2_ photosynthesis in the outdoor scaled-up reactor (50 × 30 × 5 cm^3^, 2-L working volume) with solar irradiation was performed for 5 sunny days (in May and June 2025) in the campus of Peking University (116°E, 40°N), China.

## Supplementary Material

nwaf444_Supplemental_File
