# Supplementary material for: Constructing dual charge-transfer tunnels within highly charge-confined COFs for efficient photosynthesis of hydrogen peroxide from water and air
Source: Natl Sci Rev. 2025 Oct 17;13(1):nwaf444. doi: 10.1093/nsr/nwaf444 (PMC12796805; doi:10.1093/nsr/nwaf444)
Supplement: nwaf444_Supplemental_File [file nwaf444_supplemental_file.pdf]

Supplementary Data

Constructing dual charge transfer tunnels within highly charge-confined COFs for efficient photosynthesis of hydrogen peroxide from water and air

Yanghui Hou[1,2,3], Fuyang Liu[1,2,3], Zhengmao Li[1,2,3], Jialiang Liang[4], Peng Zhou[5], and Meiping Tong[1,2,3,*]

[1] College of Environmental Sciences and Engineering, Peking University, Beijing, 100871, P. R. China.

[2] The Key Laboratory of Water and Sediment Sciences (Ministry of Education), Peking University, Beijing, 100871, P. R. China.

[3] State Environmental Protection Key Laboratory of All Material Fluxes in River Ecosystems, Peking University, Beijing, 100871, P. R. China.

[4] College of Environment and Ecology, Chongqing University, Chongqing, 400045, P. R. China.

[5] Eco-environment and Resource Efficiency Research Laboratory, School of Environment and Energy, Peking University Shenzhen Graduate School, Shenzhen, Guangdong, 518055, P. R. China.

[*] Corresponding author: Tel: +86 10 62756491; E-mail address: tongmeiping@pku.edu.cn.

NUMBER OF PAGES: 57

NUMBER OF FIGURES: 33

NUMBER OF TABLES: 6

Table of Contents

Index	Caption	Page
**Fig. S1**	XPS survey spectra of COF-APM and COF-BPM.	15
**Fig. S2**	N 1s high-resolution XPS spectra of COF-APM (a) and COF-BPM (b).	16
**Fig. S3**	Thermal gravimetric analysis of COF-APM and COF-BPM.	17
**Fig. S4**	XPS valence band spectra (a) and energy band alignment (b) of COF-APM and COF-BPM.	18
**Fig. S5**	*In-situ* irradiated N 1s XPS spectra of COF-APM (a) and COF-BPM (b).	19
**Fig. S6**	Charge distribution of excited state for long charge transfer pathway (a) and short charge transfer tunnel (b).	20
**Fig. S7**	Photocurrent responses (a) and electrochemical impedance spectra (b) of COF-APM and COF-BPM.	21
**Fig. S8**	Steady-state PL spectra (a) and fluorescence lifetime decay spectra (b) of COF-APM and COF-BPM.	22
**Fig. S9**	Temperature-dependent PL spectra of COF-APM and COF-BPM.	23
**Fig. S10**	TA spectra signals on the fs-ns timescales of COF-APM (a) and COF-BPM (b).	24
**Fig. S11**	TA decay kinetic curves of COF-APM (a) and COF-BPM (b).	25
**Fig. S12**	H_2O_2 photosynthesis kinetics by different photocatalysts under visible light irradiation.	26
**Fig. S13**	Chemical structure (a), PXRD pattern (b), SEM image (c), FT-IR spectrum (d), Hirshfeld charge analysis (e), and ESP mapping (f) of the fabricated COF-BPD.	27
**Fig. S14**	Structures of COF-1 (a), COF-PMD (b), BTT-Ph-COF (c), and BTT-MD-COF (d)	28
**Fig. S15**	Degradation of 1 mM H_2O_2 by COF-BPM under N_2 atmosphere.	29
**Fig. S16**	Dynamics of photocatalytic H_2O_2 formation and decomposition over COF-BPM at 25°C (a) and 40°C (b).	30

**Fig. S17**	Effects of COF-BPM dosage on H_2O_2 synthesis (a) and the corresponding photosynthesis kinetics (b). Effects of COF-APM dosage on H_2O_2 synthesis (c) and the corresponding photosynthesis kinetics (d).	31
**Fig. S18**	PXRD patterns (a), FT-IR spectra (b), N_2 adsorption-desorption isotherm (c), and UV-Vis DRS spectra (d) of COF-BPM before and after reaction.	32
**Fig. S19**	H_2O_2 photosynthesis kinetics by COF-BPM with different pH conditions.	33
**Fig. S20**	H_2O_2 photosynthesis kinetics by COF-BPM in different real water samples.	34
**Fig. S21**	Photosynthesis of H_2O_2 by COF-BPM on a floatable foam sheet (a) and the digital image of double wall quartz reactor with the floatable foam sheet (b).	35
**Fig. S22**	Light intensities and water temperature monitored during the the photocatalytic H_2O_2 production process by COF-BPM immobilized in the scaled-up reactor.	36
**Fig. S23**	Photographs of culture media for the disinfection of antibiotic-resistant bacteria without (a) and with (b) the mixture of H_2O_2 solution produced by COF-BPM.	37
**Fig. S24**	*In-situ* photocatalytic degradation of paracetamol by COF-BPM under visible-light irradiation.	38
**Fig. S25**	H_2O_2 photosynthesis kinetics by COF-BPM in the presence of different trapping agent under visible light irradiation.	39
**Fig. S26**	O_2 evolution by COF-BPM in the presence of $NaBrO_3$ under Ar atmosphere.	40
**Fig. S27**	ESR spectra of DMPO-·OH generated by COF-BPM.	41
**Fig. S28**	Isotopic experiment by using $H_2{}^{18}O$ as water source during H_2O_2 photosynthesis by COF-BPM.	42
**Fig. S29**	ESR spectra of DMPO-·O_2^- generated by COF-BPM.	43
**Fig. S30**	RRDE curves over COF-BPM measured at 1600 rpm in O_2-saturated electrolyte using the ring current and the disk current (a). The average number of the transferred electrons (n) at different potentials calculated from RRDE data (b).	44
**Fig. S31**	Adsorption energy of O_2 onto COF-APM and COF-BPM.	45
**Fig. S32**	Time-course *in-situ* DFTIRS of COF-APM under visible light irradiation with O_2.	46

**Fig. S33**	Mechanism of COF-BPM for dual-path photocatalytic H_2O_2 production	47
**Table S1**	Fractional atomic coordinated for unit cell of COF-BPM calculated after performing the Pawley Refinement.	48
**Table S2**	Fractional atomic coordinated for unit cell of COF-APM calculated after performing the Pawley Refinement.	49
**Table S3**	Fitted parameters and average lifetimes of fs-TA.	50
**Table S4**	Comparison of photocatalytic H_2O_2 production with other reported photocatalysts under similar measurement conditions.	51
**Table S5**	Efficiency comparison of as-prepared COFs and other reported COF photocatalysts with different charge transfer pathways.	52
**Table S6**	Characteristics of real water samples.	54

Text S1. Chemicals and reagents

5-(4-aminophenyl)pyrimidin-2-amine (APM, 97%) was purchased from Jilin Yanshen Technology Co., Ltd. 5,5'-bipyrimidine-2,2'-diamine (BPM, 97%) and 1,3,5-triformylphloroglucinol (TP, 95%) were obtained from Shanghai Bide Pharmatech Co., Ltd. NaOH and $HClO_4$ were purchased from Beijing Chemical Work. All other reagents were purchased from commercial vendors in China and used as received without further purification.

Text S2. Characterizations

The crystalline structure of COFs was determined by powder X-ray diffraction (PXRD, X-Pert3 Powder, PANalytical, Netherlands) employing Cu Kα (λ = 0.154 nm) irradiation at 40 kV and 40 mA ranging from 2θ = 2° up to 40° with 0.02° increment. The solid-state ^{13}C cross-polarization magic angle spinning nuclear magnetic resonance spectroscopy (^{13}C CP/MAS NMR) of COF-APM and COF-BPM were recorded by an Advance III spectrometer (Bruker, Switzerland). The Fourier transform infrared (FT-IR) spectra of COFs were recorded by a Nicolet is50 FT-IR spectrometer (ThermoFisher, USA). The XPS analyses of COFs were determined by an Axis Ultra (Kratos Analytical Ltd., UK) photoelectron spectrometer using a monochromatic Al Kα line source. The C1s peak at 284.8 eV was used to calibrate the position of other peaks. The Brunauer–Emmett–Teller (BET) specific surface areas of COFs were obtained using N_2 isotherms at 77K with an ASAP2460 instrument (Micromeritics Instrument Ltd., USA). Thermal gravimetric analyses (TGA) were performed on a Q600 SDT analyzer (TA Instruments, USA) after further drying (at 80°C under air for 12 h), with a temperature range from 25 to 600°C and a heating rate of 10°C min^{-1} in N_2 atmosphere. The water adsorption analysis of COFs was performed at 298 K by using a 3Flex (Micromeritics Instrument Ltd., USA). The morphology of COF-APM and COF-BPM was determined using a field-emission scanning electron microscope (FE-SEM, S-4800, HITACHI, Japan) and a transmission electron microscope (TEM, Tecnai G2 F30, FEI, USA). Time-resolved PL spectroscopy of COFs was

recorded on a FLS980 spectrophotometer (Edinburgh, UK). Temperature-dependent PL spectra from 158K to 298K were also recorded on a FLS980 spectrophotometer (Edinburgh, UK). The TA spectroscopy was conducted by using a commercial TA spectrometer (Helios, Ultrafast System, USA) with an excitation source of a femtosecond-pulsed amplifier (Astrella, 80 fs, 1 kHz, Coherent), which was doubled by the Coherent Libra regenerative amplifier (800 nm 80 fs, 1 kHz). The surface potential images and signals of COFs were measured using KPFM (Bruker) under ambient atmosphere in the amplitude-modulated (AM-KPFM) mode. During the measurement of the surface potential, the lift mode was used with a lift height of 20 nm. In the lift mode, the topography and the surface potential signals were sequentially recorded.

The ultraviolet-visible diffuse reflectance spectra (UV-Vis DRS) were recorded for the dry pressed disk COFs samples using a UV3600PLUS spectrophotometer (Shimadzu, Japan) with $BaSO_4$ as a reference. The optical bandgap (E_g) was calculated according to following equation [S1]:

$$(\alpha \cdot hv)^{1/n} = B(hv\text{-}E_g) \qquad \text{(Eq. S1)}$$

where α is the adsorption coefficient, h is the Planck constant, v is the light frequency, B is a constant and E_g is the corresponding bandgap. In addition, the n is determined by the nature of the electron transition [S2].

Text S3. Photocatalytic experiments

The photosynthesis of H_2O_2 was first performed in a double wall quartz reactor with 40 mL of ultrapure water and 5 mg of COF-APM and COF-BPM. The initial solution pH of the mixture was adjusted by NaOH or $HClO_4$. The reaction suspension was irradiated by using a 300 W Xenon lamp (λ > 420 nm, 1000 ± 10 $W \cdot m^{-2}$). The temperature was fixed at 25.0 ± 0.2°C by circulating water system during the photocatalytic experiments. The reaction suspension was extracted and filtered for H_2O_2 measurement at specific time intervals. For the experiments investigating the effects of pH on H_2O_2 photosynthesis by COF-BPM, the pH value of suspension was set from 3 to 11, in which either 0.1 M $HClO_4$ or 0.1 M NaOH solution was used to adjust the solution pH. The reusability of COF-BPM toward H_2O_2 production in five consecutively used

cycles were also testified. The photocatalytic generation of H_2O_2 in real water samples including tap water, lake water, river water, and sea water was examined. Details about four real waters were exhibited in **Table S6**. All error bars represent the standard deviation and are calculated on the basis of two independent experiments.

To immobilize COF-BPM onto a floatable foam sheet, a 2×2×0.2 cm^3 dried sponge with a density of 40 mg cm^{-3} was placed at the bottom of Teflon-lined stainless-steel autoclave during the typical fabrication process of COF-BPM. In the continuous-flow experiment, 20 mg powder of COF-BPM was dispersed in a mixture of ethanol (4.5 mL) and Nafion (0.5 mL, 5% v/v), and then coated on the surface of channel in the continuous-flow reactor. The fabricated continuous-flow reactor was operated for 30 h with ~1 mL min^{-1} flow rate. To further determine the feasibility of COF-BPM for H_2O_2 photosynthesis in the practical applications under natural sunlight irradiation, COF-BPM was immobilized in a scaled-up reactor (50 cm×30 cm×5 cm) with working volume of 2 L water, and then the immobilized sample was performed outdoors. Specifically, the homogeneous suspension containing 200 mg of COF-BPM powder, 18 mL ethanol and 2 mL Nafion was dropped onto the surface (50 cm×30 cm) of scaled-up reactor and then dried in air to obtain the immobilized COF-BPM. Its corresponding photocatalytic production toward H_2O_2 were evaluated in a larger reactor with working volume of 2 L outdoors with natural sunlight irradiation on sunny days (May and June 2025) in the campus of Peking University (116ºE, 40ºN), Beijing. During the outdoor experiments, both the temperature of water and intensities of sunlight were regularly monitored. The immobilized COF-BPM in the scaled-up reactor was repeated used for five days.

Text S4. Measurement of H_2O_2

The concentration of H_2O_2 was determined by iodometry. Specifically, 1 mL of samples was added to the mixture of 1 mL of 0.4 M KI and 1 mL 0.1 M potassium hydrogen phthalate ($C_8H_5KO_4$), which was kept for 1 h. Under acidic conditions, H_2O_2 can react with I^- to generate I_3^- ($H_2O_2 + 3I^- + 2H^+ \rightarrow I_3^- + 2H_2O$), which

exhibits strong absorption at approximately 350 nm. Thus, the absorbance at 350 nm by using UV-vis spectroscopy can measure the amount of I_3^-, which can further determine the amount of H_2O_2 produced in each sample.

Text S5. Kinetics analysis of H_2O_2

Kinetic analysis was carried out to assess the formation and decomposition rate constants of H_2O_2 during the photocatalysis by COF-BPM at both 25 and 40°C. The kinetic constants were calculated by fitting the H_2O_2 production curves via the following Box-Lucas model [S3].

$$[H_2O_2]=\left(\frac{K_f}{K_d}\right)\times(1-e^{-K_d t}) \quad \text{(Eq. S2)}$$

where K_f is the H_2O_2 formation rate constant, K_d is the H_2O_2 decomposition rate constant, t is the reaction time, and $[H_2O_2]$ is the concentration of produced H_2O_2.

Text S6. Determination of SCC efficiency

The photocatalytic reaction was conducted in ultrapure water (2 mL) with COF-BPM (3 mg) in a round-bottomed Pyrex glass flask with magnetic stirring. After O_2 bubbling, the suspension was irradiated by an Xe lamp with light intensity of 100 mW cm^{-2}. Typically, the SCC efficiency was calculated by using the following equation:

$$SCC(\%)=\frac{[\triangle G \text{ for } H_2O_2 \text{ generation (J mol}^{-1})][H_2O_2 \text{ formed (mol)}]}{[\text{total input power (W)}][\text{reaction time (s)}]}\times 100\% \quad \text{(Eq. S3)}$$

where $\triangle G$= 117 kJ mol^{-1}. In this study, the irradiated area is 1×10^{-4} m^2, and the light intensity of Xenon lamp is 100 mW cm^{-2}. Thus, the total input power is calculated to be 0.1 W.

Text S7. Determination of apparent quantum yield (AQY)

The photocatalytic reaction system of AQY was conducted in ultrapure water (2 mL) with COF-BPM (3 mg) in a round-bottomed Pyrex glass flask (irradiated area: 1×10^{-4} m^2) with magnetic stirring. After O_2

bubbling, the suspension was irradiated by the specific wavelength light. The intensity of incident light at specific wavelength is 20.9 (purple light at 400 nm), 48.2 (blue light at 462 nm), 36.3 (green light at 522 nm), 32.2 (yellow light at 575 nm), and 34.1 W m^{-2} (red light at 626 nm), respectively. The AQY of COF-BPM at specific wavelength could be calculated by the following equation [S4]:

$$AQY(\%) = \frac{2 \times H_2O_2 \text{ formed (mol)}}{\text{the number of incident photons (mol)}} \times 100\% = \frac{n_P \times N_A \times h \times c}{P \times S \times t \times \lambda} \times 100\% \qquad \text{(Eq. S4)}$$

where n_p is the mole number of product obtained (mol), N_A is the Avogadro constant (6.022×10^{23} mol^{-1}), P is the optical density (W m^{-2}), S is the light irradiation area (m^2), t is the light irradiation time (s), λ is the monochromatic light wavelength (m), *h* is Planck's constant (6.626×10^{-34} J s), and *c* is the speed of light (3×10^8 m s^{-1}).

Text S8. Structure modeling and theoretical calculations

The structure information of COF-APM and COF-BPM (using the hexagonal crystal cell) was simulated by Materials Studio software [S5]. The Hirshfeld charge distribution of obtained models was investigated by CESTEP software package using the generalized gradient approximation Perdew-Burke-Ernzenhof (GGA-PBE) method with energy cutoff of 400 eV and energy convergence of 1.0×10^{-5} eV. The brillouin zone is sampled with 2 × 2 × 1 Gamma mesh [S6]. Test calculations show that this cutoff and k-mesh are sufficient to obtain converged energies. The surface electrostatic potential and excited-state charge distribution of COF-APM and COF-BPM (using the repeating unit) was performed on Gaussian 16 software package using the B3LYP functional at the hybrid 6-31(d, p) method [S7]. The adsorption energy (E_{ads}) of O_2 on COFs can be calculated by using following equations:

$$E_{ads} = E_{total} - (E_{substrate} + E_{adsorbate}) \qquad \text{(Eq. S5)}$$

where E_{total}, $E_{substrate}$, and $E_{adsorbate}$ are the total energies of the adsorption system, substrate (COFs), and free adsorbate (O_2), respectively.

The electronic structure of fabricated COFs was investigated by the Vienna Ab-initio Simulation Package

(VASP) using the Perdew-Burke-Ernzerhof (PBE) of the generalized gradient approximation (GGA). The PAW pseudo-potential of N ($2s^22p^3$), C ($2s^22p^2$), O ($2s^22p^4$) and H ($1s^1$) potentials were employed to describe the interaction between valence electrons and the ionic core. The geometry optimization of COFs was conducted by a plane-wave basis with energy cutoff of 400 eV and an energy convergence threshold of 1.0×10^{-4} eV at the gamma point. The vacuum layer between two neighboring layers of COFs was set to 15 Å, which was used to exclude the interaction between neighboring COFs layers. The Monkhorst-Pack k-point meshes are 2 × 2 × 1 for COF-APM and COF-BPM. The calculations of Gibbs free energy changes (ΔG) of all reaction steps adopted the known standard hydrogen electrode (SHE) model. The G was calculated by the following formula:

$$G = E + H(T) - TS \quad \text{(Eq. S6)}$$

where E, H(T) and S are the electronic free energy, enthalpy and entropy of model at T = 298.15K, respectively. The highest reaction energy of elemental steps was used as the energy barrier in each pathway.

Text S9. Details of the photocatalytic degradation experiment

In the paracetamol photocatalytic degradation experiment, 5 mg of COF-BPM were added into 40 mL paracetamol solution with an initial concentration of 5 mg L^{-1}. Prior to the photocatalytic experiments, the reaction suspension was firstly stirred under dark condition for 30 min to reach adsorption-desorption equilibrium. At decided time intervals, approximately 1 mL of suspension was sampled and filtrated with a 0.22 μm membrane filter to remove photocatalyst particles. The residual concentration of paracetamol in suspension was determined by high-performance liquid chromatography (HPLC, Agilent U3000), equipped with an Agilent Zorbax SB-Aq column (4.6 mm × 250 mm, 5 μm).

Text S10. Details of bacterial cultivation, counting and photocatalytic experiments

Kanamycin -resistant *E. coli* was cultivated in 100 mL growth medium, containing 10 g L^{-1} tryptone,

10 g L^{-1} NaCl and 5 g L^{-1} bacto-yeast extract. *B. subtilis* was also cultivated in 100 mL growth medium, containing 15 g L^{-1} tryptone, 5 g L^{-1} NaCl and 5 g L^{-1} bacto-yeast extract. The flask with the growth media were shaken at 200 revolutions per minute in an incubator (37 °C and 16 h for *E. coli*, 30 °C and 16 h for *B. subtilis*). Bacterial cells were harvested by centrifugation at 5000 rpm for 8 min. The growth media were then discarded after the centrifugation. Subsequently, the bacterial pellets were washed with sterilized physiological saline (0.9% of NaCl solution at pH 7.0) for three times to remove the residual growth medium. The cell precipitates were then re-dispersed in sterilized physiological saline with certain volumes to obtain the bacterial stock suspensions. The viable cell density of the bacterial stock suspension was typically ~10^9 colony forming unit per milliliter (CFU mL^{-1}).

For the *ex-situ* photocatalytic disinfection experiment, the produced H_2O_2 was directly mixed with Kanamycin -resistant *E. coli* (with viable cell density of 1×10^3 CFU mL^{-1} in suspension) for 24 h. For the *in-situ* photocatalytic disinfection experiment, 5 mg of COF-BPM was well dispersed in 49.8 mL sterilized saline with 15 mM NaCl under sonication. Certain amount of bacteria stock suspension was added into COFs suspension to set the target viable cell density of 10^5 CFU mL^{-1}. The suspensions were then magnetically stirring for 5 min prior to the visible light irradiation. During the disinfection process, 0.5 mL bacterial suspension was sampled at given intervals and serially diluted with sterilized deionized water. After that, 0.1 mL of the diluted samples were immediately spread on nutrient agar plates and incubated at the temperature of 37°C for 24 h. After that, 0.1 mL of the diluted samples were then spread on nutrient agar plates and incubated at the temperature of 37°C for 24 h.

Text S11. Photocatalytic H_2O_2 production with different trapping agents and atmospheres

In quenching experiments, tert-butyl alcohol (TBA, 1 mmolL^{-1}), methanol (MeOH, 1 mmolL^{-1}), and *p*-benzoquinone (*p*-BQ, 0.1 mmolL^{-1}) were employed to explore the contribution of diffusing OH, h^+, and O_2^- in the reaction system, respectively. For the H_2O_2 production under N_2 atmospheres, the solution

was bubbled with N_2 for 30 min before reaction, and continuous N_2 bubbling during the photocatalysis process. Other reaction conditions and process are the same with photocatalytic H_2O_2 production experiment.

Text S12. Details of ESR analysis

The generation of reactive species including photogenerated electrons (e^-), superoxide radicals (O_2^-), and hydroxyl radicals (OH) by COF-BPM during the H_2O_2 photosynthesis process were investigated by ESR measurements. 5,5-dimethyl-1-pyrroline N-oxide (DMPO) and 4-hydroxy-2,2,6,6-tetramethylpiperidinyloxy (TEMPO) were used as the spin trapping agents. 1 μL DMPO was added into 50 μL sample suspension (1 g/L in DMSO) for the detection of O_2^-. 1 μL DMPO was added into 100 μL sample suspension (1 g L^{-1} in water) for the detection of OH. Additionally, 1 μL TEMPOL (2.5 mM) was added into 200 μL sample suspension (1 g L^{-1} in water) for the detection of e^-. The mixed suspensions were placed into the quartz capillary tube and then irradiated by visible-light to detect the signal intensity of the spin capture adduct.

Text S13. Determination of concentrations of $\cdot O_2^-$

Nirtoblue tetrazolium (NBT, 1.25×10^{-5} M, with an absorption maximum at 259 nm) was employed as probe molecules to determine the amount of O_2^- generated in the photocatalytic system. Typically, 1 mL NBT stock solution was introduced into our photocatalytic system. Suspension was collected at different time intervals and filtrated by 0.22 μm membrane to exclude the influence of photocatalysts for the determination of O_2^- concentration with UV-Vis spectrophotometer (Shimadzu, UV-1780).

Text S14. Photoelectrochemical measurements

All photoelectrochemical measurements were performed on a CHI 760E (CH Instruments Ins.) electrochemical workstation. A three-electrode cell system was employed including a reference electrode of Ag/AgCl, a counter electrode of Pt foil, and a working electrode. For fabrication of the working electrode,

the 5 mg powder of samples was first dispersed in a mixture of ethanol (450 μL) and Nafion (50 μL, 5% v/v), and then coasted on a 1.0 × 2.0 cm^2 ITO glass. 0.1 M Na_2SO_4 solution was used as the electrolyte. The transient photocurrents response was conducted under visible-light irradiation. The visible light was obtained from a 300 W xenon lamp with a UV cutoff filter (λ ⩾ 420 nm). The light intensity was adjusted to be 100 $mW\cdot cm^{-2}$. The electrochemical impedance spectra (EIS) were collected with the frequency range between 1×10^6 Hz to 1 Hz at an AC amplitude of 20 mV. Rotating ring-disk electrode (RRDE) analysis was conducted in a three-electrode cell by using Pt foil as a counter electrode and using Ag/AgCl as a reference electrode, respectively. The RRDE was consist of a glassy carbon disk and Pt ring. Before the experiment, COF-BPM was dropped onto the glassy carbon disk and then dried.

Text S15. ^{18}O isotopic experiment

$H_2{}^{18}O$ with saturated $^{16}O_2$ was employed to replace water in a sealed reactor for H_2O_2 production by COF-BPM, which was irradiated by Xenon lamp (>420 nm). After H_2O_2 photosynthesis, the reaction suspension was purged by N_2 to remove $^{16}O_2$ in the reactor. Subsequently, the photo-generated H_2O_2 was decomposed into O_2 by adding catalase. The generated O_2 gas was analyzed by a gas chromatography-mass spectrometry (GC-MS, Agilent 7890B-5977B).

Text S16. Details for *in-situ* DFTIRS measurement

In-situ Fourier transform infrared spectrometry was conducted on a Nicolet is50 FT-IR spectrometer (ThermoFisher, USA). The photocatalysts were filled into an *in-situ* IR cell in a chamber. Before the measurement, the chamber was degassed under Ar flow at 393 K for 2 h. The baseline was then obtained at room temperature. 5 μL H_2O was added onto the samples with the continuous Ar flow through the chamber. Subsequently, 10 mL of O_2 was directly injected into the sealed chamber, followed by the visible light irradiation through the window of the chamber. The *in-situ* DFTIRS were recorded at specific intervals.

Text S17. Reaction equations

$$\text{COF-BPM} \rightarrow e^{-} + h^{+} \quad \text{(Eq. S7)}$$

$$H_2O + h^{+} \rightarrow {}^{*}OH + H^{+} \quad \text{(Eq. S8)}$$

$$2\ {}^{*}OH \rightarrow H_2O_2 \quad \text{(Eq. S9)}$$

$$O_2 + e^{-} \rightarrow \cdot O_2^{-} \quad \text{(Eq. S10)}$$

$$\cdot O_2^{-} + e^{-} + 2H^{+} \rightarrow H_2O_2 \quad \text{(Eq. S11)}$$

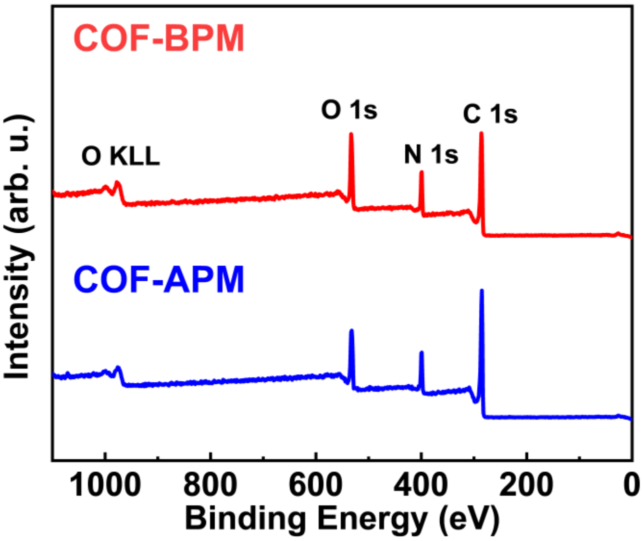

Figure S1. XPS survey spectra of COF-APM and COF-BPM.

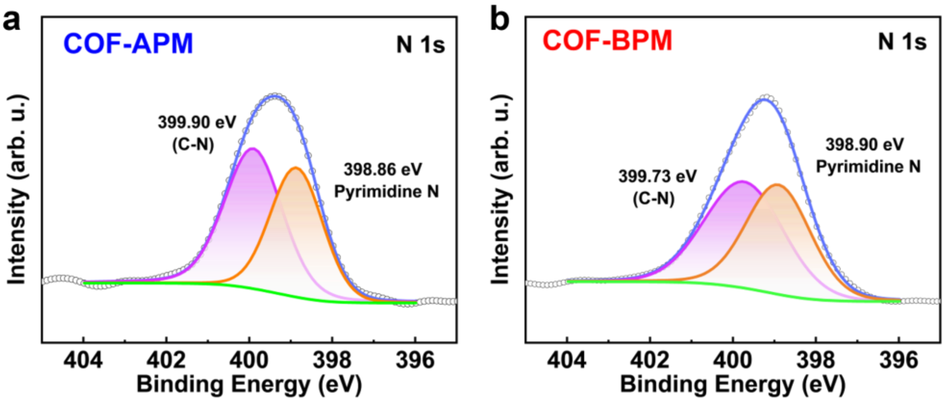

Figure S2. N 1s high-resolution XPS spectra of COF-APM (a) and COF-BPM (b).

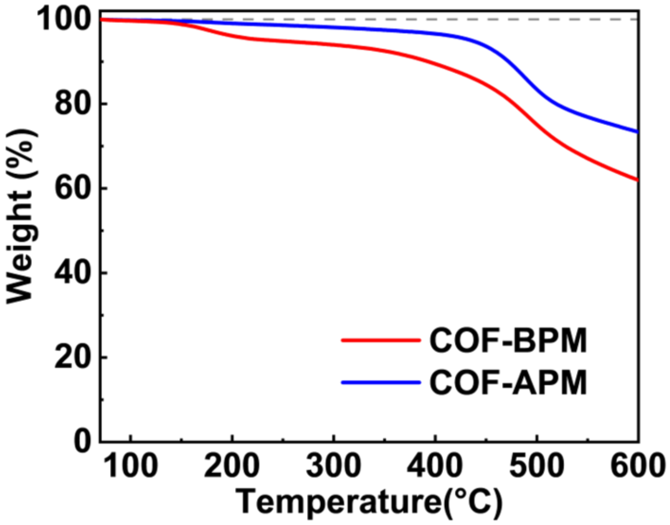

Figure S3. Thermal gravimetric analysis of COF-APM and COF-BPM.

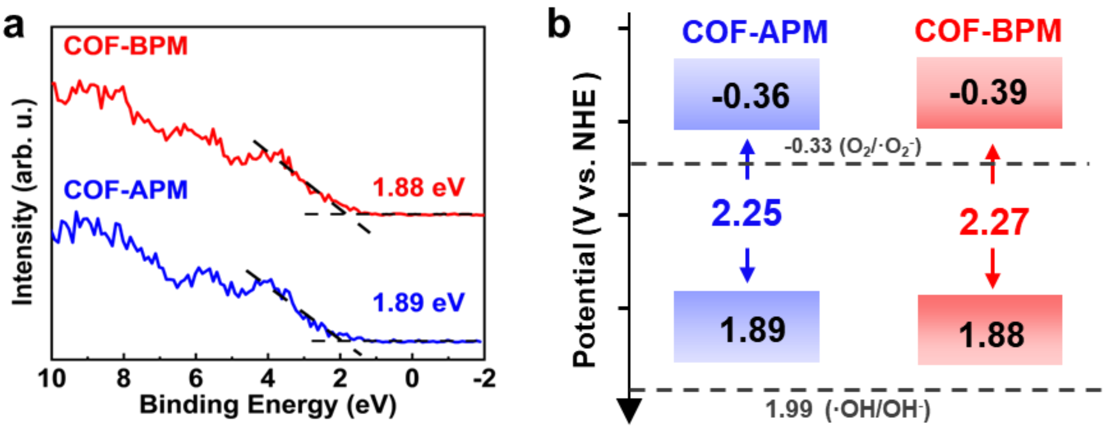

Figure S4. XPS valence band spectra (a) and energy band alignment (b) of COF-APM and COF-BPM.

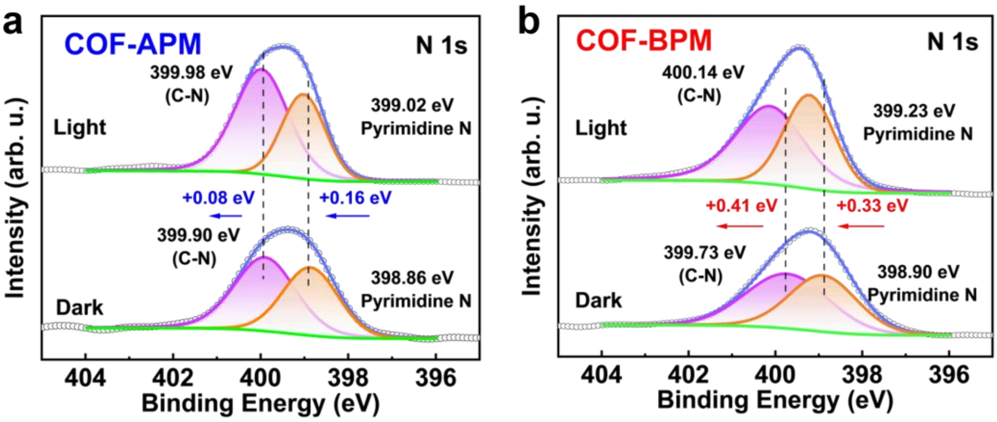

Figure S5. *In-situ* irradiated N 1s XPS spectra of COF-APM (a) and COF-BPM (b).

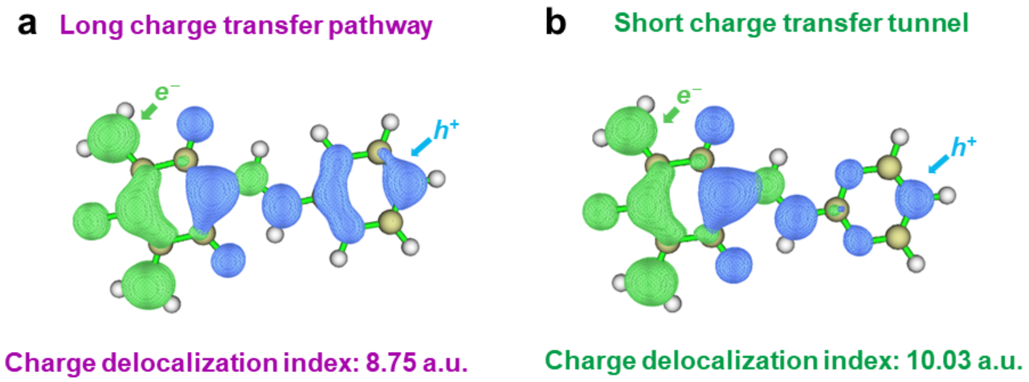

Figure S6. Charge distribution of excited state for long charge transfer pathway (a) and short charge transfer tunnel (b).

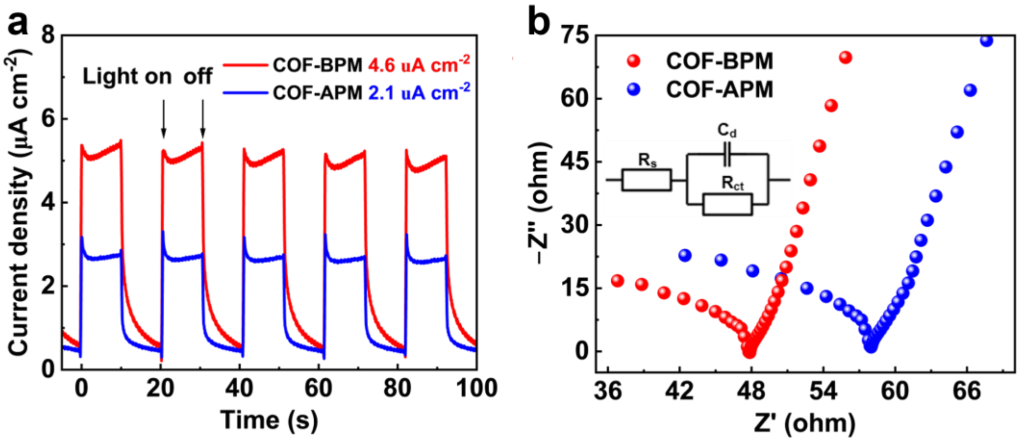

Figure S7. Photocurrent responses (a) and electrochemical impedance spectra (b) of COF-APM and COF-BPM.

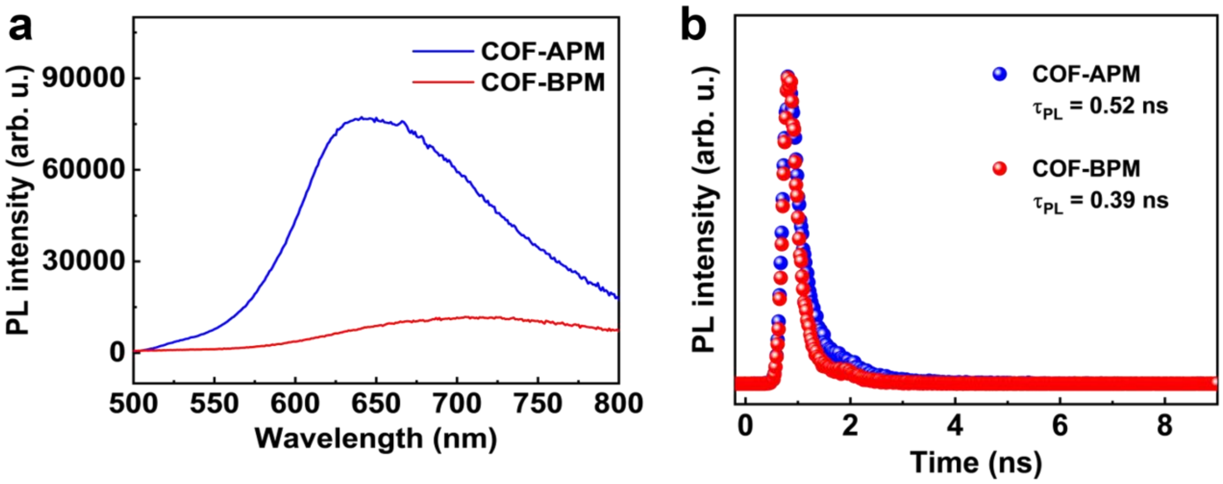

Figure S8. Steady-state PL spectra (a) and fluorescence lifetime decay spectra (b) of COF-APM and COF-BPM.

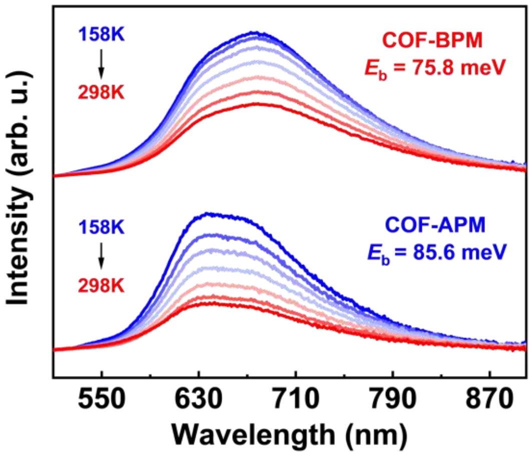

Figure S9. Temperature-dependent PL spectra of COF-APM and COF-BPM.

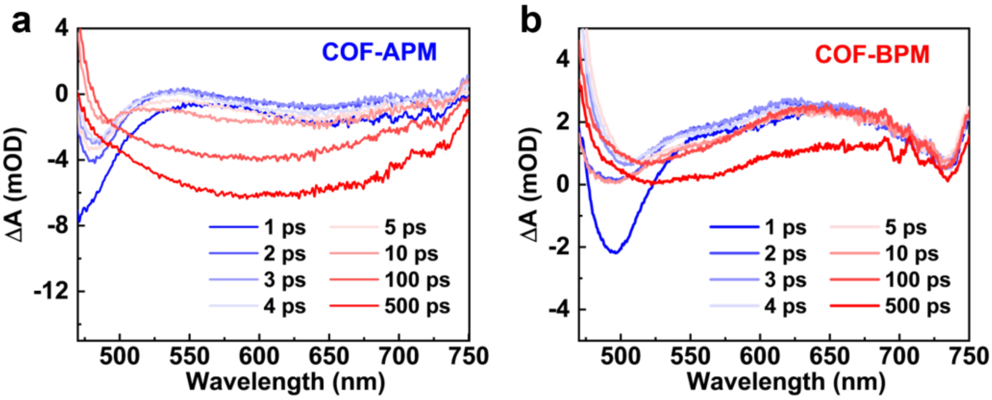

Figure S10. TA spectra signals on the fs-ns timescales of COF-APM (a) and COF-BPM (b).

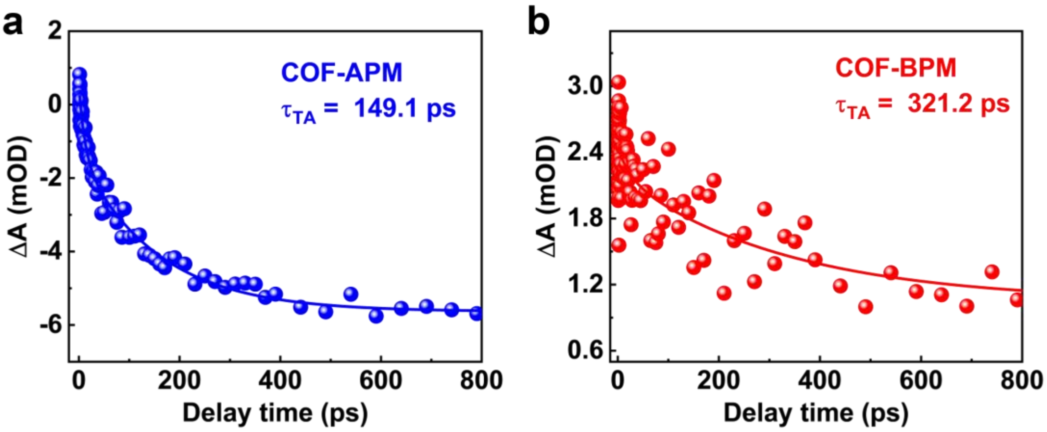

Figure S11. TA decay kinetic curves of COF-APM (a) and COF-BPM (b).

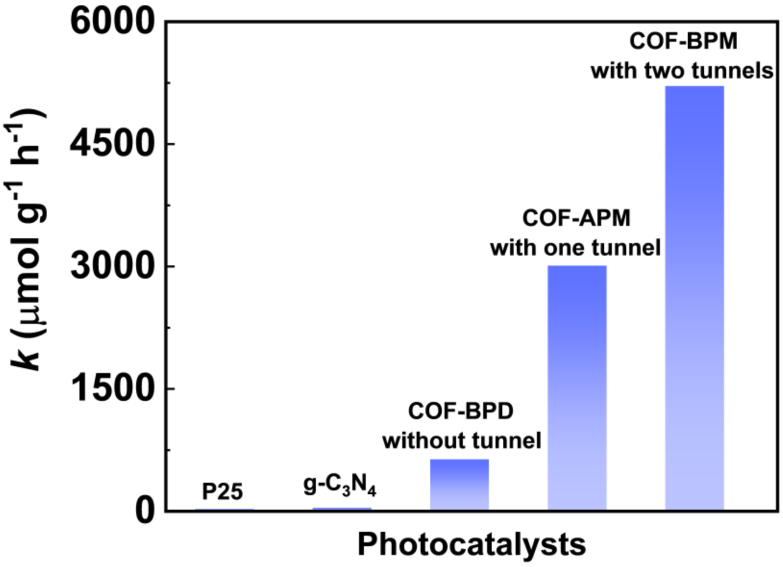

Figure S12. H_2O_2 photosynthesis kinetics by different photocatalysts under visible light irradiation.

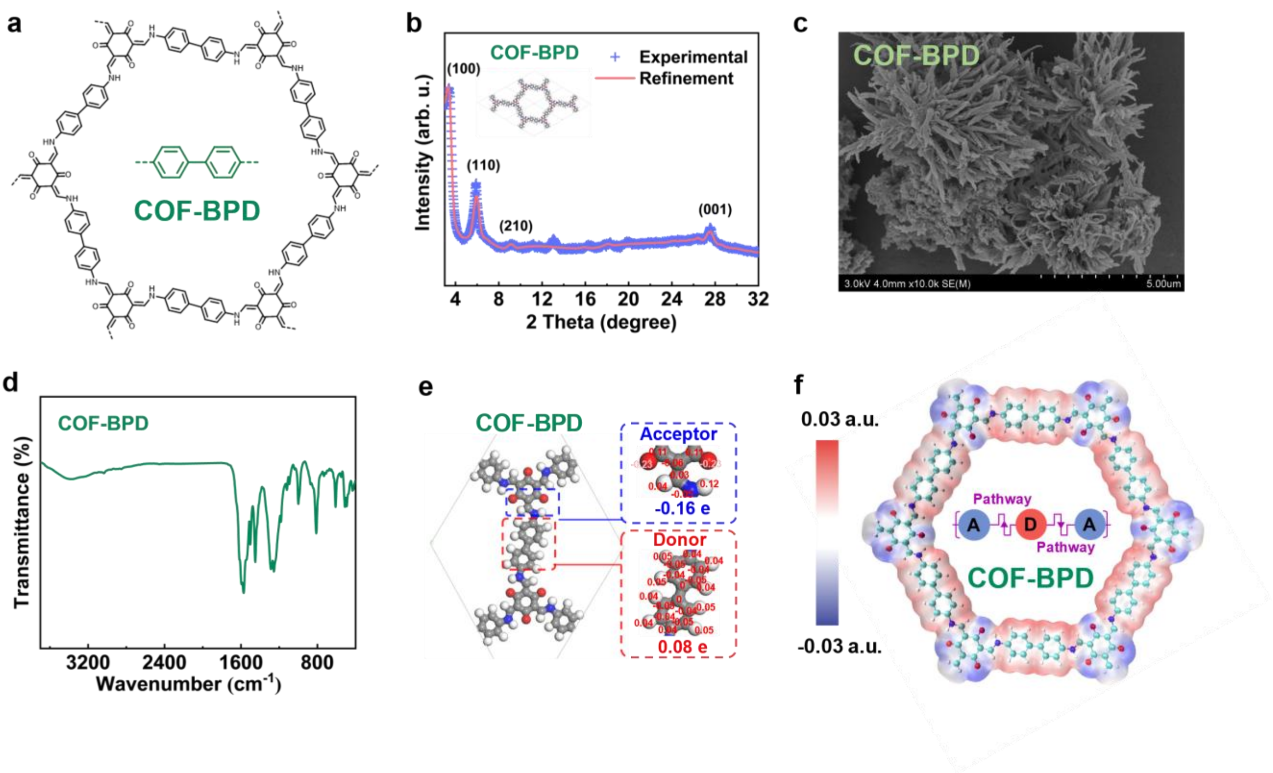

Figure S13. Chemical structure (a), PXRD pattern (b), SEM image (c), FT-IR spectrum (d), Hirshfeld charge analysis (e), and ESP mapping (f) of the fabricated COF-BPD.

The biphenyl-based COF-BPD was synthesized via a one-pot solvothermal method. The mixture containing 4,4'-biphenylenediamine (BPD, 0.6 mmol, 110.5 mg), TP (0.4 mmol, 84.1 mg), Mesitylene (4 mL), 1,4-dioxane (4 mL), and 6 M HAc (2 mL) was sonicated in a 20 mL Teflon lining. The mixture was ultrasonicated and bubbled with N_2 for 20 min. Following that, the mixture was heated at 120°C for 72 h. The formed precipitate was washed with acetone for three times and dried at 60°C under air for 12 h to obtain COF-0CN. The PXRD pattern (**Figure S13b**), SEM image (**Figure S13c**), and FT-IR spectrum (**Figure S13d**) of the fabricated COF-BPD indicated the successful synthesis of the designed framework structure. Both the Hirshfeld charge analysis (**Figure S13e**) and the ESP mapping (**Figure S13f**) suggest that COF-BPD contains two long charge transfer pathways yet without charge transfer tunnel.

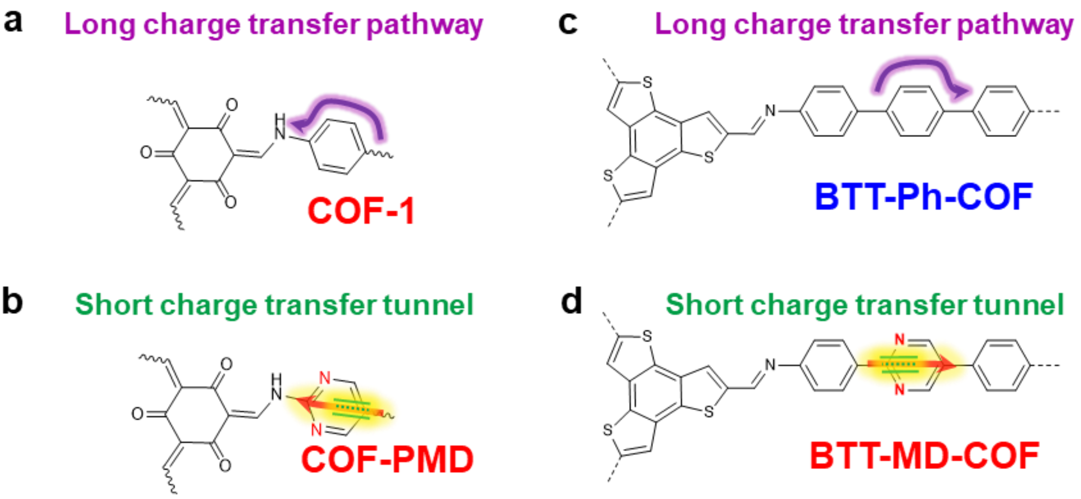

Figure S14. Structures of COF-1 (a), COF-PMD (b), BTT-Ph-COF (c), and BTT-MD-COF (d), the insets are corresponding charge transfer pathways.

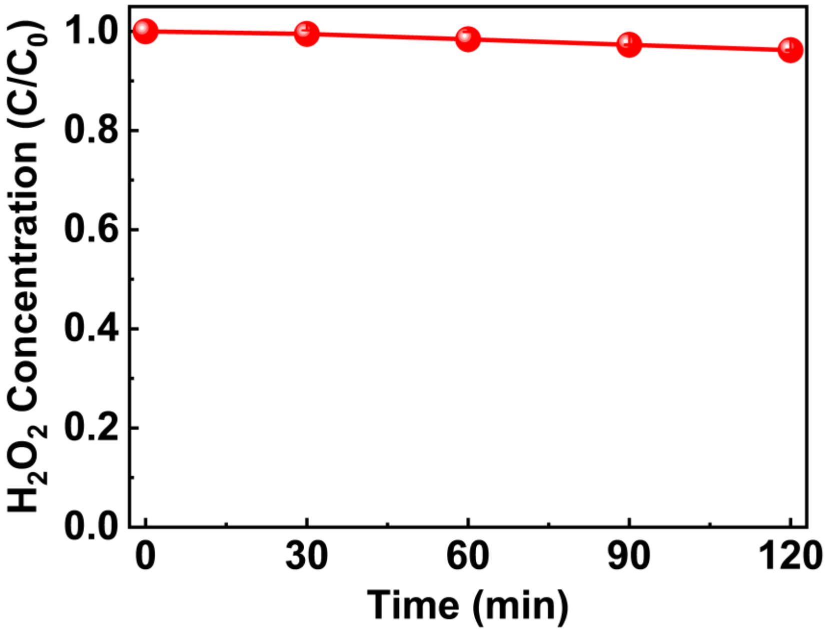

Figure S15. Degradation of 1 mM H_2O_2 by COF-BPM under N_2 atmosphere.

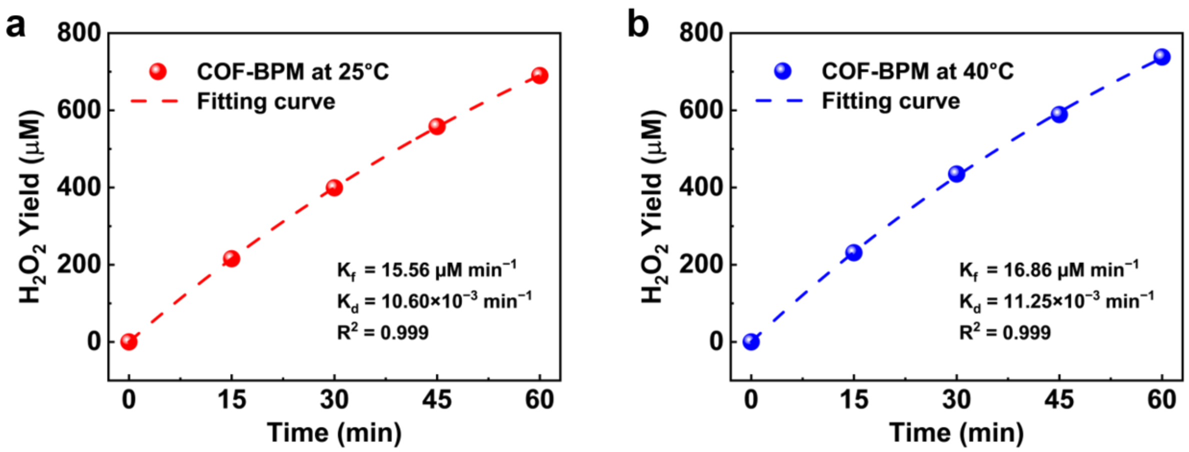

Figure S16. Dynamics of photocatalytic H_2O_2 formation and decomposition over COF-BPM at 25℃ (a) and 40℃ (b).

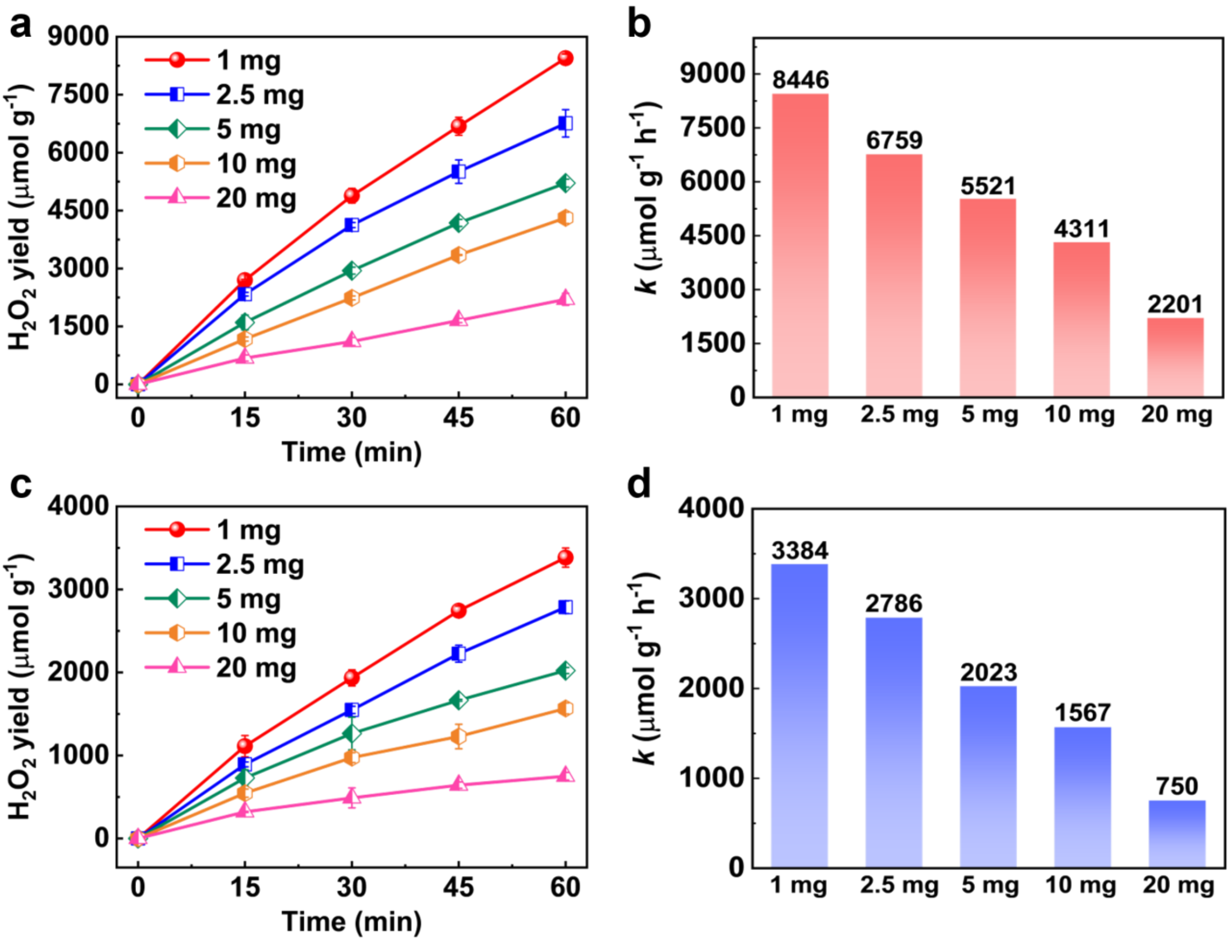

Figure S17. Effects of COF-BPM dosage on H_2O_2 synthesis (a) and the corresponding photosynthesis kinetics (b). Effects of COF-APM dosage on H_2O_2 synthesis (c) and the corresponding photosynthesis kinetics (d).

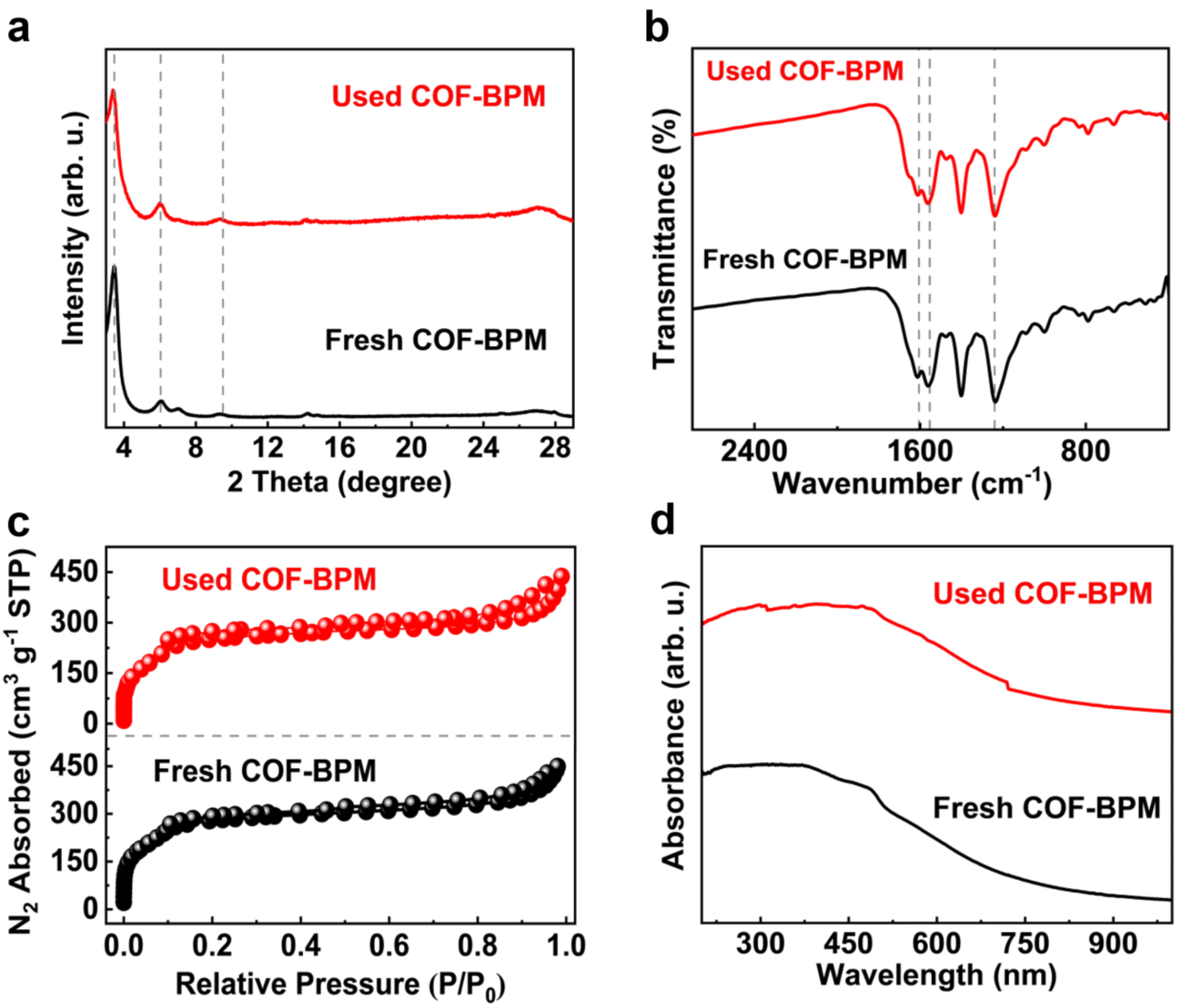

Figure S18. PXRD patterns (a), FT-IR spectra (b), N_2 adsorption-desorption isotherm (c), and UV-Vis DRS spectra (d) of COF-BPM before and after reaction.

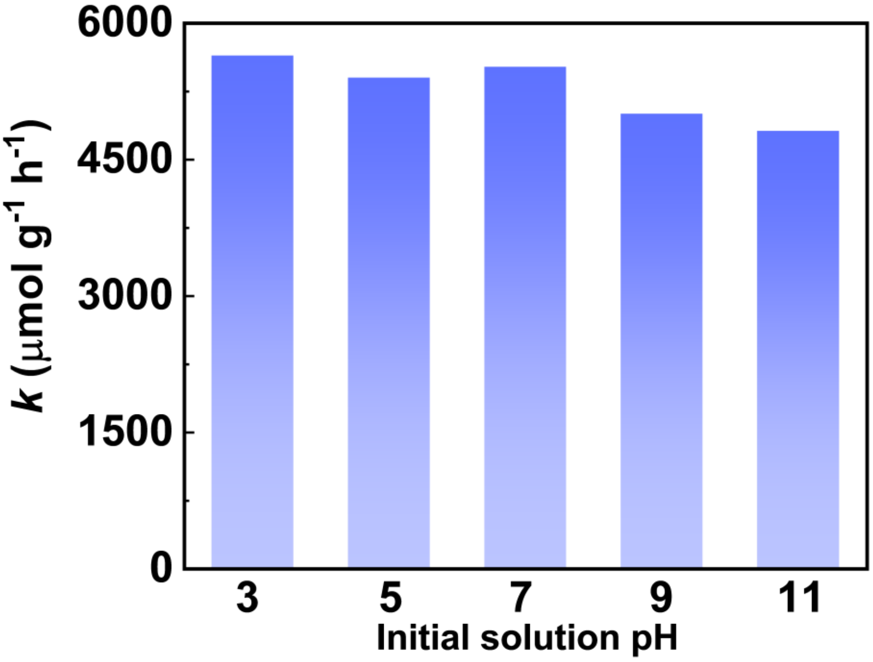

Figure S19. H_2O_2 photosynthesis kinetics by COF-BPM with different pH conditions.

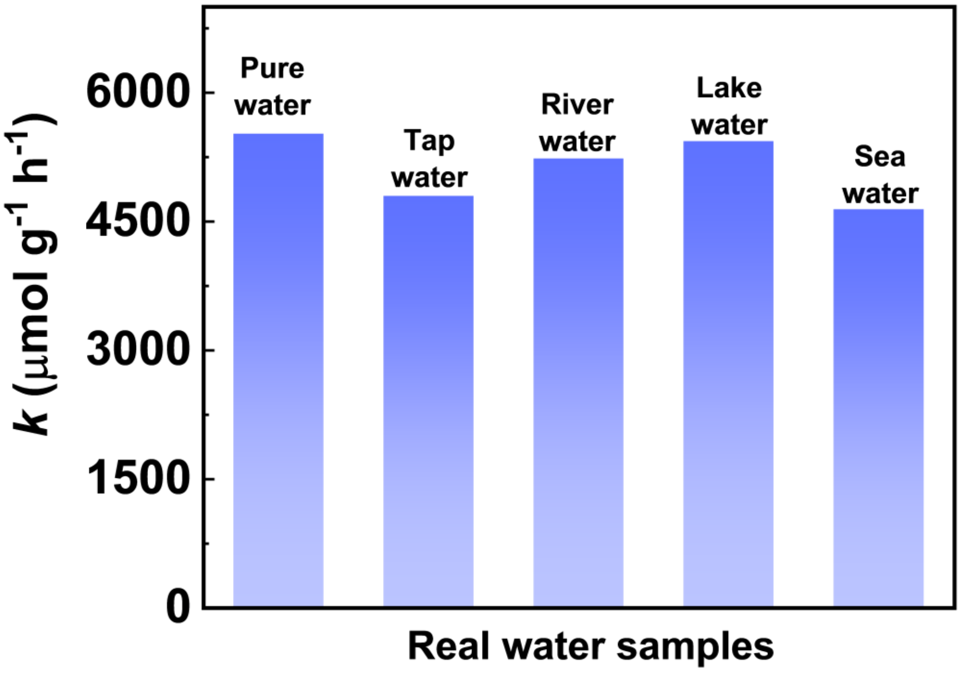

Figure S20. H_2O_2 photosynthesis kinetics by COF-BPM in different real water samples.

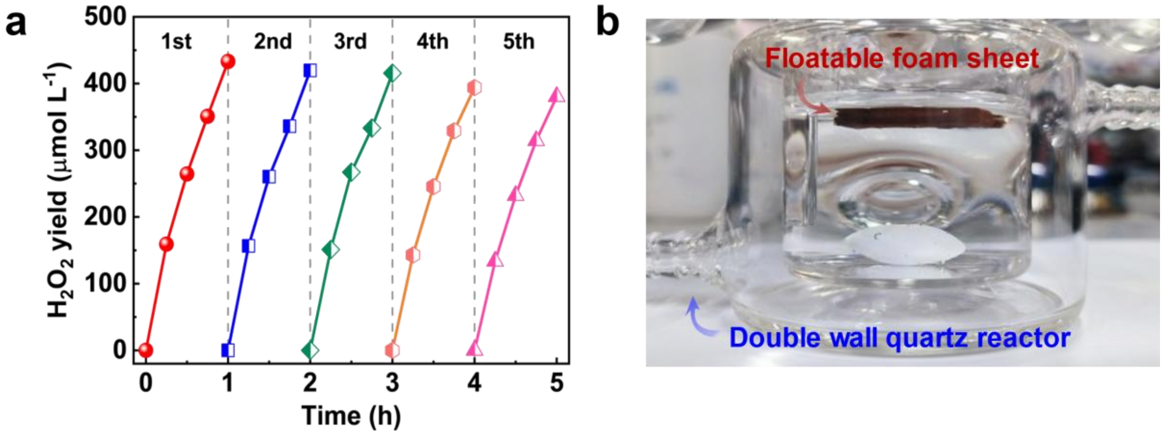

Figure S21. Photosynthesis of H_2O_2 by COF-BPM on a floatable foam sheet (a) and the digital image of double wall quartz reactor with the floatable foam sheet (b).

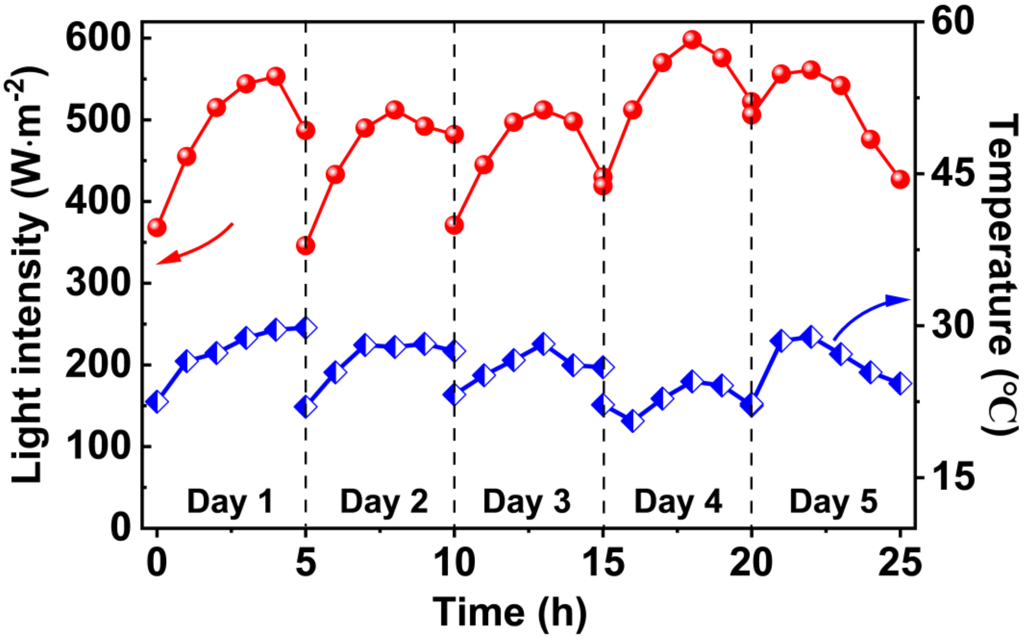

Figure S22. Light intensities and water temperature monitored during the the photocatalytic H_2O_2 production process by COF-BPM immobilized in the scaled-up reactor.

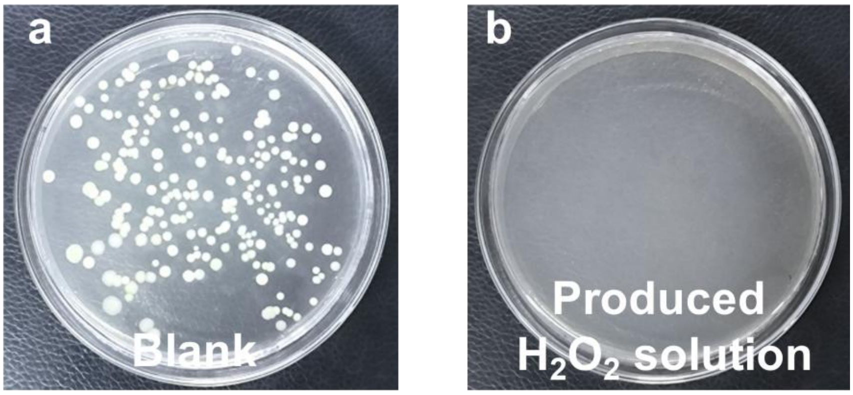

Figure S23. Photographs of culture media for the disinfection of antibiotic-resistant bacteria without (a) and with (b) the mixture of H_2O_2 solution produced by COF-BPM.

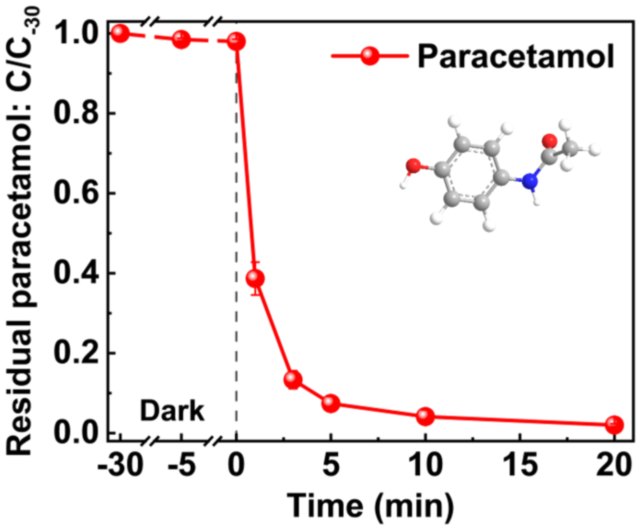

Figure S24. *In-situ* photocatalytic degradation of paracetamol by COF-BPM under visible-light irradiation.

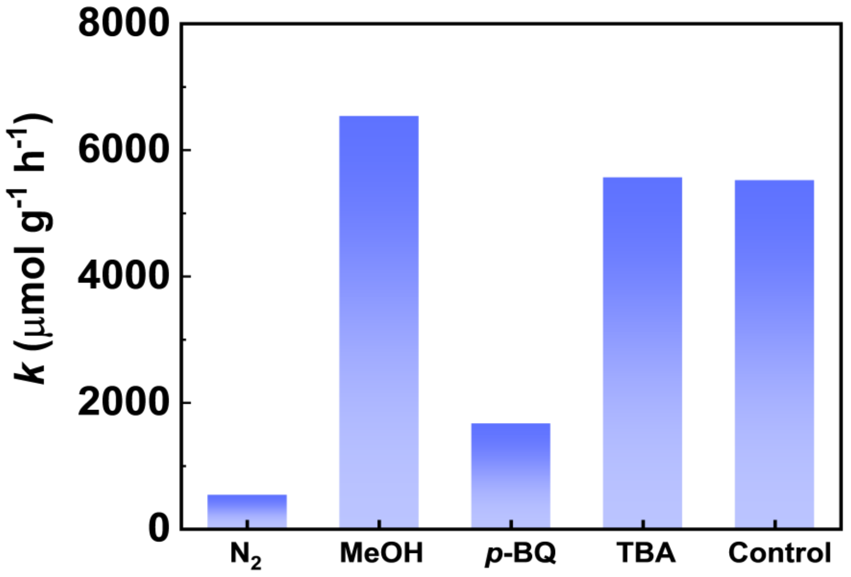

Figure S25. H_2O_2 photosynthesis kinetics by COF-BPM in the presence of different trapping agent under visible light irradiation.

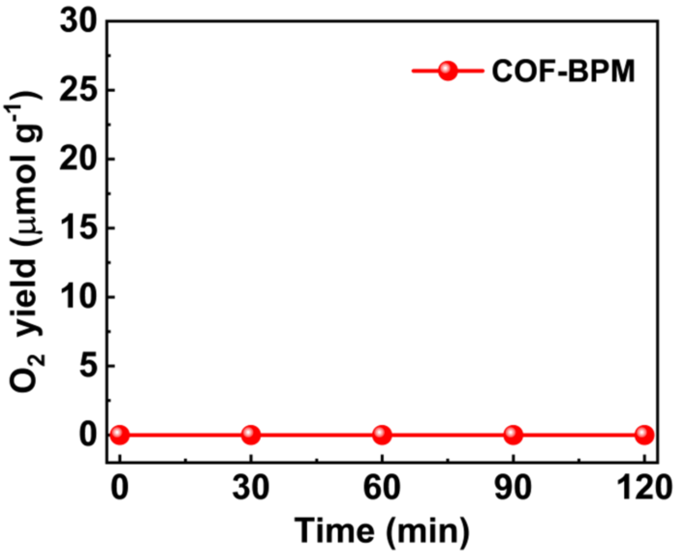

Figure S26. O_2 evolution by COF-BPM in the presence of $NaBrO_3$ under Ar atmosphere.

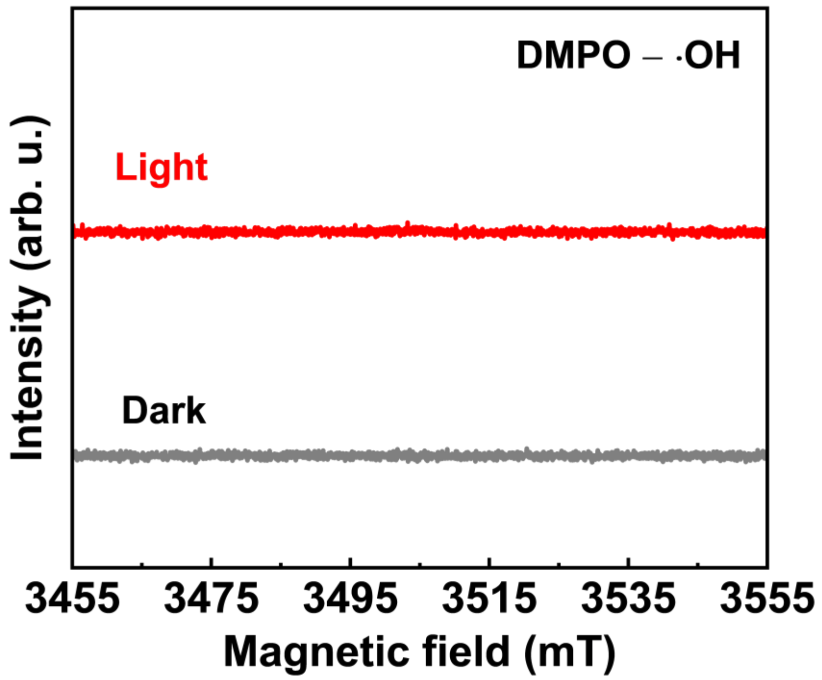

Figure S27. ESR spectra of DMPO-·OH generated by COF-BPM.

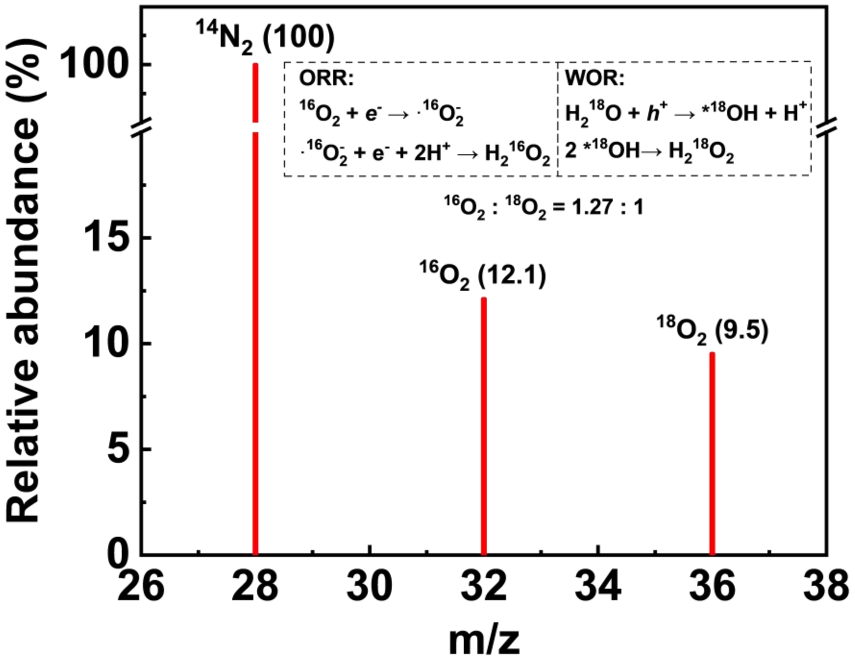

Figure S28. Isotopic experiment by using $H_2{}^{18}O$ as water source during H_2O_2 photosynthesis by COF-BPM.

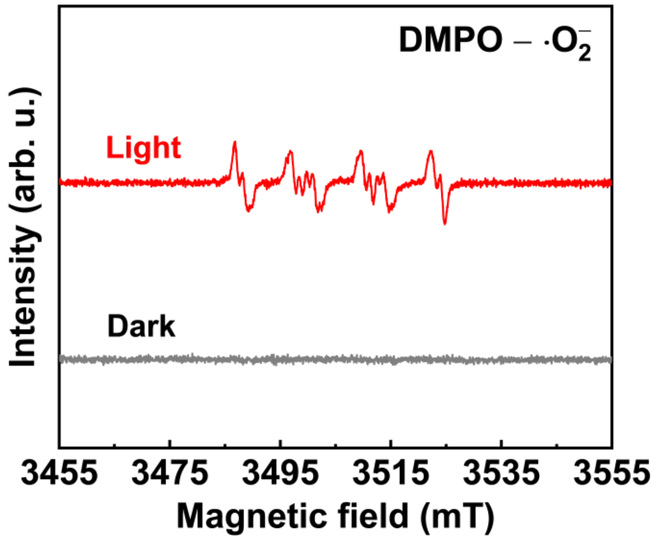

Figure S29. ESR spectra of DMPO-·O_2^- generated by COF-BPM.

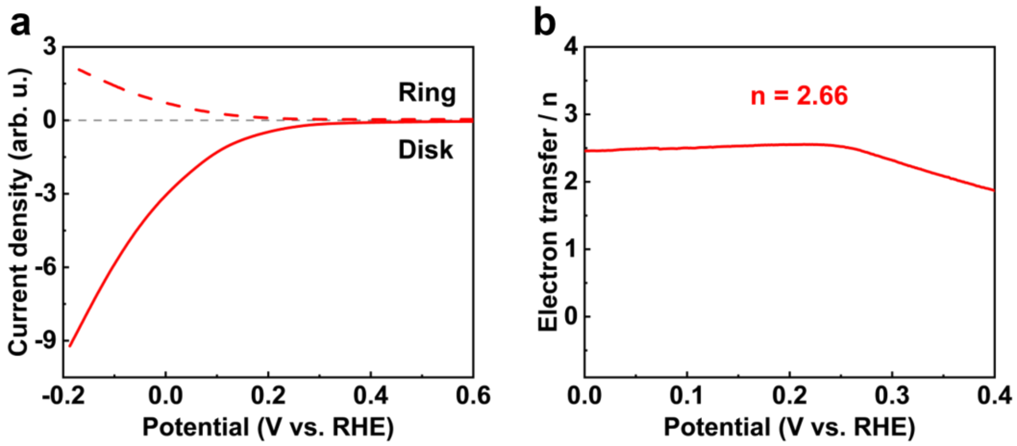

Figure S30. RRDE curves over COF-BPM measured at 1600 rpm in O_2-saturated electrolyte using the ring current and the disk current (a). The average number of the transferred electrons (n) at different potentials calculated from RRDE data (b).

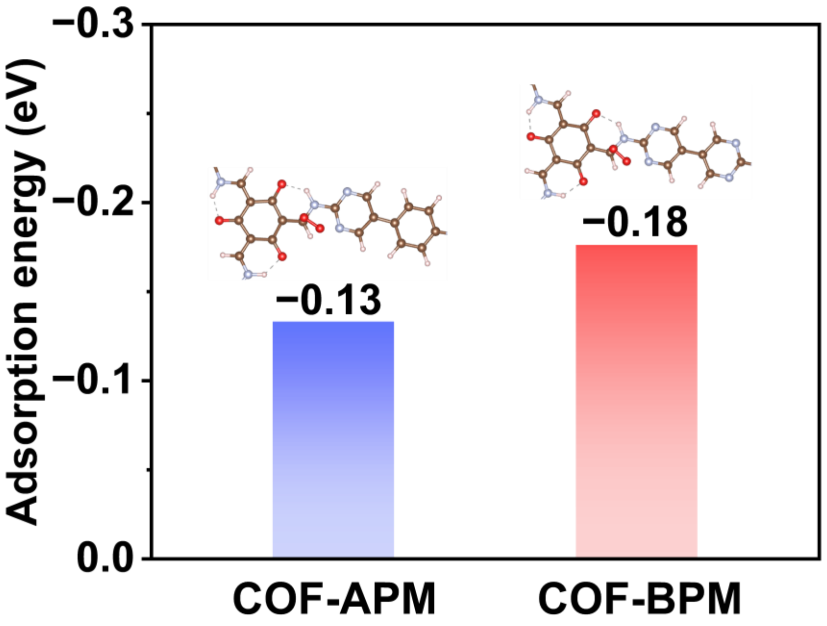

Figure S31. Adsorption energy of O_2 onto COF-APM and COF-BPM.

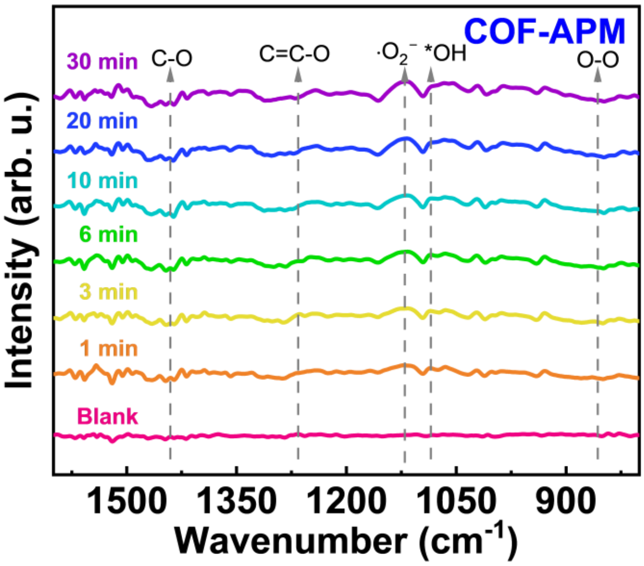

Figure S32. Time-course *in-situ* DFTIRS of COF-APM under visible light irradiation with O_2.

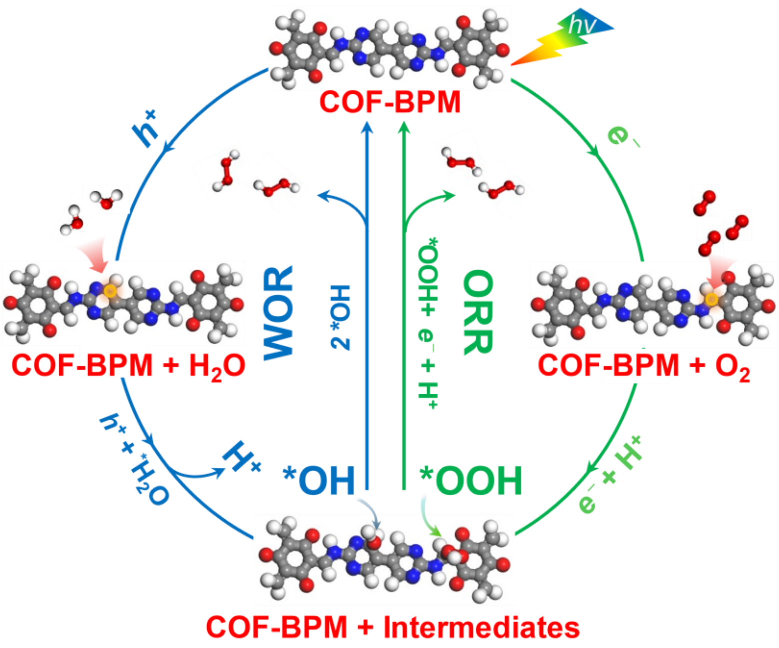

Figure S33. Mechanism of COF-BPM for dual-path photocatalytic H_2O_2 production, where gray, blue, red, white, and orange spheres represent C, N, O, H atoms and active sites, respectively.

Table S1. Fractional atomic coordinated for unit cell of COF-BPM calculated after performing the Pawley Refinement**.**

**Space group**	P6		
**Calculated cell parameters**	a = b = 29.14 Å, c = 3.34 Å, α = β = 90°, γ = 120°, R_{wp} = 3.04%, R_p = 2.27%		
**Atoms**	x	y	z
C1	0.72413	0.36021	0.5
C2	0.69667	0.3897	0.5
O3	0.72077	0.43609	0.5
C4	0.77604	0.3818	0.5
N5	0.81303	0.43558	0.5
C6	0.86681	0.4537	0.5
N7	0.88479	0.42071	0.5
C8	0.93554	0.43741	0.5
C9	0.97158	0.49012	0.5
C10	0.95135	0.52325	0.5
N11	0.90022	0.50453	0.5
H12	0.7917	0.35658	0.5
H13	0.8034	0.46397	0.5
H14	0.94652	0.40869	0.5
H15	0.97515	0.56406	0.5
C16	0.27587	0.63979	0.5
C17	0.30333	0.6103	0.5
O18	0.27923	0.56391	0.5
C19	0.22396	0.6182	0.5
N20	0.18697	0.56442	0.5
C21	0.13319	0.5463	0.5
N22	0.11521	0.57929	0.5
C23	0.06446	0.56259	0.5
C24	0.02842	0.50988	0.5
C25	0.04865	0.47675	0.5
N26	0.09978	0.49547	0.5
H27	0.2083	0.64342	0.5
H28	0.1966	0.53603	0.5
H29	0.05348	0.59131	0.5
H30	0.02485	0.43594	0.5
H30	0.02485	0.43594	0.5

Table S2. Fractional atomic coordinated for unit cell of COF-APM calculated after performing the Pawley Refinement.

**Space group**	P6		
**Calculated cell parameters**	a = b = 28.18 Å, c = 3.28 Å, α = β = 90°, γ = 120°, R_{wp} = 3.44%, R_p = 2.53%		
**Atoms**	x	y	z
C1	0.72406	0.36341	0.5
C2	0.6946	0.39125	0.5
O3	0.71734	0.44005	0.5
C4	0.7756	0.38743	0.5
N5	0.81128	0.44314	0.5
C6	0.86599	0.46069	0.5
C7	0.88424	0.42341	0.5
C8	0.93572	0.43926	0.5
C9	0.9725	0.49294	0.5
C10	0.95542	0.53154	0.5
C11	0.90218	0.51541	0.5
H12	0.89014	0.54619	0.5
H13	0.79033	0.36108	0.5
H14	0.80037	0.47236	0.5
H15	0.86264	0.38054	0.5
H16	0.94414	0.40698	0.5
H17	0.98086	0.57429	0.5
C18	0.27594	0.63659	0.5
C19	0.3054	0.60875	0.5
O20	0.28266	0.55995	0.5
C21	0.2244	0.61257	0.5
N22	0.18872	0.55686	0.5
C23	0.13401	0.53931	0.5
N24	0.11576	0.57659	0.5
C25	0.06428	0.56074	0.5
C26	0.0275	0.50706	0.5
C27	0.04458	0.46846	0.5
N28	0.09782	0.48459	0.5
H29	0.20967	0.63892	0.5
H30	0.19963	0.52764	0.5
H31	0.05586	0.59302	0.5
H32	0.01914	0.42571	0.5

148 **Table S3.** Fitted parameters and average lifetimes of fs-TA.

Sample	τ_{TA}^{*} (ps)	τ_1 (ps)	R_1 (%)	τ_2 (ps)	R_2 (%)
COF-APM	149.1	12.2	4.4	155.4	95.6
COF-BPM	321.2	23.9	2.6	329.2	97.4

149 $^{*}\tau_{TA} = (R_1 \times \tau_1^2 + R_2 \times \tau_2^2)/(R_1 \times \tau_1 + R_2 \times \tau_2)$.

150

Table S4. Comparison of photocatalytic H_2O_2 production with other reported photocatalysts under similar measurement conditions.

Catalyst	Dosage (mg)	Time (h)	H_2O_2 yield (μmol g^{-1} h^{-1})	Reaction conditions	Reference
COF-BPM	5	1	5521	Solution volume: 50 mL<br>Xe lamp (λ≥ 420 nm):<br>100 mW·cm^{-2}<br>O_2 saturated pure water	This work
COF-APM	5	1	2023	Solution volume: 50 mL<br>Xe lamp (λ≥ 420 nm):<br>100 mW·cm^{-2}<br>O_2 saturated pure water	This work
COF-BPD	5	1	631	Solution volume: 50 mL<br>Xe lamp (λ≥ 420 nm):<br>100 mW·cm^{-2}<br>O_2 saturated pure water	This work
CN-PDI-rGO	50	24	23	Solution volume: 30 mL<br>Xe lamp (λ≥420 nm):<br>43.3 mW·cm^{-2}<br>O_2 saturated pure water	[S9]
OCN-500	50	10	106	Solution volume: 50 mL<br>Xe lamp (λ≥420 nm):<br>35 mW·cm^{-2}<br>O_2 saturated pure water	[S10]
R_{370}-CN	100	1	170	Solution volume: 100 mL<br>Xe lamp (λ≥420 nm)<br>O_2 saturated pure water	[S11]
CTF-BDDBN	30	24	146	Solution volume: 50 mL<br>Xe lamp (λ≥420 nm):<br>45 mW·cm^{-2}<br>O_2 saturated pure water	[S12]
COF-JLU51	5	1	4260	Solution volume: 50 mL<br>Xe lamp (λ≥420 nm):<br>100 mW·cm^{-2}<br>O_2 saturated pure water	[S13]
Nv-C≡N-CN	20	1	137	Solution volume: 20 mL<br>Xe lamp (λ≥420 nm):<br>40 mW·cm^{-2}<br>O_2 saturated pure water	[S14]

COF-TfpBpy	15	0.67	695	Solution volume: 10 mL<br>Xe lamp (λ≥420 nm):<br>401 mW·cm^{-2}<br>pure water	[S15]
TPE-AQ	10	1	909	Solution volume: 20 mL<br>Xe lamp (λ≥400 nm):<br>100 mW·cm^{-2}<br>Pure water	[S16]
sonoCOF-F2	3	1.5	1250	Solution volume: 5 mL<br>Xe lamp (λ≥420 nm)<br>O_2 saturated pure water	[S17]
TaptBtt	15	1.5	1407	Solution volume: 10 mL<br>O_2 saturated pure water	[S18]
FS-COFs	5	1	1502	Solution volume: 20 mL<br>LED lamp (λ≥400 nm)<br>O_2 saturated pure water	[S19]
COF-2CN	1	2	4858	Solution volume: 50 mL<br>Xe lamp (λ≥ 420 nm):<br>100 mW·cm^{-2}<br>O_2 saturated pure water	[S20]
TAPT-FTPB COFs	1	1.25	3780	Solution volume: 10 mL<br>AM 1.5G<br>O_2 saturated pure water	[S21]
PB-COF	15	1	2044	Solution volume: 20 mL<br>AM 1.5G<br>325 mW·cm^{-2}<br>O_2 saturated pure water	[S22]
TACOF-1-COOH	2.5	1	3542	Solution volume: 18 mL<br>Xe lamp (λ≥ 420 nm):<br>O_2 saturated pure water	[S23]
COF-BD2	5	1	5211	Solution volume: 50 mL<br>Xe lamp (λ≥ 420 nm):<br>100 mW·cm^{-2}<br>O_2 saturated pure water	[S24]

Table S5. Efficiency comparison of as-prepared COFs and other reported COF photocatalysts with different charge transfer tunnel.

Num.	Sample#1[*]	Sample#2[**]	Ratio of performance[***]	Reference
1	COF-1	COF-PMD	17.4	[S2]
2	BTT-Ph-COF	BTT-MD-COF	1.94	[S8]

[*]The COFs without charge transfer tunnel

[**]The COFs with single charge transfer tunnel

[***]Ratio of performance corresponds to the ratio of photocatalytic performance of Sample#1 to that of Sample#2 in the article.

Table S6. Characteristics of real water samples.

Index (mg/L)	Tap Water (TW)	River Water (RW)	Lake Water (LW)	Sea Water (SW)
pH	7.5	7.7	6.9	7.6
TOC	0.9	4.7	8.1	121.7
NO_3^-	15.5	32.7	6.4	Not Detected
Cl^-	32.7	100.7	49.4	18886
SO_4^{2-}	95.0	77.7	107.6	2102
Na^+	25.2	55.2	35.4	9001
K^+	1.1	11.9	2.6	251.8
Mg^{2+}	31.0	20.0	37.1	1147

Reference

S1. Makuła P, Pacia M, Macyk W. How to correctly determine the band gap energy of modified semiconductor photocatalysts based on uv-vis spectra. *J Phys Chem Lett*. 2018; **9**(23): 6814-6817.

S2. Liu F, Ma Z, Deng Y *et al.* Tunable covalent organic frameworks with different heterocyclic nitrogen locations for efficient Cr(VI) reduction, Escherichia coli disinfection, and paracetamol degradation under visible-light irradiation. *Environ Sci Technol*. 2021; **55**(8): 5371-5381.

S3. Zhang X, Cheng S, Chen C *et al.* Keto-anthraquinone covalent organic framework for H_2O_2 photosynthesis with oxygen and alkaline water. *Nat Commun*. 2024; **15**(1): 2649.

S4. Shen R, Huang C, Hao L *et al.* Ground-state charge transfer in single-molecule junctions covalent organic frameworks for boosting photocatalytic hydrogen evolution. *Nat Commun*. 2025; **16**(1): 2457.

S5. Li D, Li C, Zhang L *et al.* Metal-free thiophene-sulfur covalent organic frameworks: Precise and controllable synthesis of catalytic active sites for oxygen reduction. *J Am Chem Soc*. 2020; **142**(18): 8104-8108.

S6. Li C, Liu J, Li H *et al.* Covalent organic frameworks with high quantum efficiency in sacrificial photocatalytic hydrogen evolution. *Nat Commun*. 2022; **13**(1): 2357.

S7. Wu Y, Ji H, Liu Q *et al.* Visible light photocatalytic degradation of sulfanilamide enhanced by Mo doping of BiOBr nanoflowers. *J Hazard Mater*. 2022; **424**: 127563.

S8. Chen H, Zhang H, Chi K *et al.* Pyrimidine-containing covalent organic frameworks for efficient photosynthesis of hydrogen peroxide via one-step two electron oxygen reduction process. *Nano Res*. 2024; **17**(11): 9498-9506.

S9. Kofuji Y, Isobe Y, Shiraishi Y *et al.* Carbon nitride aromatic diimide–graphene nanohybrids: Metal-free photocatalysts for solar-to-hydrogen peroxide energy conversion with 0.2% efficiency. *J Am Chem Soc*. 2016; **138**(31): 10019-10025.

S10. Wei Z, Liu M, Zhang Z *et al.* Efficient visible-light-driven selective oxygen reduction to hydrogen

peroxide by oxygen-enriched graphitic carbon nitride polymers. *Energ Environ Sci*. 2018; **11**(9): 2581-2589.

S11. Zhu Z, Pan H, Murugananthan M *et al.* Visible light-driven photocatalytically active g-C_3N_4 material for enhanced generation of H_2O_2. *Appl Catal B Environ*. 2018; **232**: 19-25.

S12. Chen L, Wang L, Wan Y *et al.* Acetylene and diacetylene functionalized covalent triazine frameworks as metal-free photocatalysts for hydrogen peroxide production: A new two-electron water oxidation pathway. *Adv Mater*. 2020; **32**(2): 1904433.

S13. Zhang Z, Zhang Q, Hou Y *et al.* Tris(triazolo)triazine-based covalent organic frameworks for efficiently photocatalytic hydrogen peroxide production. *Angew Chem Int Ed*. 2024; **63**(45): e202411546.

S14. Zhang X, Ma P, Wang C *et al.* Unraveling the dual defect sites in graphite carbon nitride for ultra-high photocatalytic H_2O_2 evolution. *Energ Environ Sci*. 2022; **15**(2): 830-842.

S15. Kou M, Wang Y, Xu Y *et al.* Molecularly engineered covalent organic frameworks for hydrogen peroxide photosynthesis. *Angew Chem Int Ed*. 2022; **61**(19): e202200413.

S16. Ye Y-X, Pan J, Shen Y *et al.* A solar-to-chemical conversion efficiency up to 0.26% achieved in ambient conditions. *Proc Natl Acad Sci*. 2021; **118**(46): e2115666118.

S17. Zhao W, Yan P, Li B *et al.* Accelerated synthesis and discovery of covalent organic framework photocatalysts for hydrogen peroxide production. *J Am Chem Soc*. 2022; **144**(22): 9902-9909.

S18. Qin C, Wu X, Tang L *et al.* Dual donor-acceptor covalent organic frameworks for hydrogen peroxide photosynthesis. *Nat Commun*. 2023; **14**(1): 5238.

S19. Luo Y, Zhang B, Liu C *et al.* Sulfone-modified covalent organic frameworks enabling efficient photocatalytic hydrogen peroxide generation via one-step two-electron O_2 reduction. *Angew Chem Int Ed*. 2023; **62**(26): e202305355.

S20. Hou Y, Zhou P, Liu F *et al.* Efficient photosynthesis of hydrogen peroxide by cyano-containing covalent organic frameworks from water, air and sunlight. *Angew Chem Int Ed*. 2024; **63**(6): e202318562.

S21. Liu Y, Li L, Sang Z *et al.* Enhanced hydrogen peroxide photosynthesis in covalent organic

frameworks through induced asymmetric electron distribution. *Nat Synth*. 2024.

S22. Chi W, Liu B, Dong Y *et al.* Boosting H_2O_2 photosynthesis by accumulating photo-electrons on carbonyl active site of polyimide covalent organic frameworks. *Appl Catal B Environ*. 2024; **355**: 124077.

S23. Xu H, Wang Y, Xu Y *et al.* Integrating multipolar structures and carboxyl groups in sp^2-carbon conjugated covalent organic frameworks for overall photocatalytic hydrogen peroxide production. *Angew Chem Int Ed*. 2024; **63**(41): e202408802.

S24. Hou Y, Liu F, Liang J *et al.* Building a confluence charge transfer pathway in COFs for highly efficient photosynthesis of hydrogen peroxide from water and air. *Angew Chem Int Ed*. 2025; **64**(24): e202505621.
